# Comprehensive Evaluation of Healthy Volunteers Using Multi-Modality Brain Injury Assessments: An Exploratory, Observational Study

**DOI:** 10.3389/fneur.2018.01030

**Published:** 2018-12-17

**Authors:** Lindell K. Weaver, Steffanie H. Wilson, Anne S. Lindblad, Susan Churchill, Kayla Deru, Robert Price, Christopher S. Williams, William W. Orrison, Jigar B. Patel, James M. Walker, Anna Meehan, Susan Mirow, the NORMAL Study Team

**Affiliations:** ^1^Division of Hyperbaric Medicine Intermountain Medical Center, Murray, UT, United States; ^2^Intermountain LDS Hospital, Salt Lake City, UT, United States; ^3^Department of Internal Medicine, School of Medicine, University of Utah, Salt Lake City, UT, United States; ^4^The Emmes Corporation, Rockville, MD, United States; ^5^Evans Army Community Hospital, Fort Carson, CO, United States; ^6^Lovelace Biomedical Research, Albuquerque, NM, United States

**Keywords:** neurological evaluation, healthy volunteers, neuroepidemiology, white matter hyperintensities, brain imaging, mild traumatic brain injury

## Abstract

**Introduction:** Even though mild traumatic brain injury is common and can result in persistent symptoms, traditional measurement tools can be insensitive in detecting functional deficits after injury. Some newer assessments do not have well-established norms, and little is known about how these measures perform over time or how cross-domain assessments correlate with one another. We conducted an exploratory study to measure the distribution, stability, and correlation of results from assessments used in mild traumatic brain injury in healthy, community-dwelling adults.

**Materials and Methods:** In this prospective cohort study, healthy adult men and women without a history of brain injury underwent a comprehensive brain injury evaluation that included self-report questionnaires and neurological, electroencephalography, sleep, audiology/vestibular, autonomic, visual, neuroimaging, and laboratory testing. Most testing was performed at 3 intervals over 6 months.

**Results:** The study enrolled 83 participants, and 75 were included in the primary analysis. Mean age was 38 years, 58 were male, and 53 were civilians. Participants did not endorse symptoms of post-concussive syndrome, PTSD, or depression. Abnormal neurological examination findings were rare, and 6 had generalized slowing on electroencephalography. Actigraphy and sleep diary showed good sleep maintenance efficiency, but 21 reported poor sleep quality. Heart rate variability was most stable over time in the sleep segment. Dynavision performance was normal, but 41 participants had abnormal ocular torsion. On eye tracking, circular, horizontal ramp, and reading tasks were more likely to be abnormal than other tasks. Most participants had normal hearing, videonystagmography, and rotational chair testing, but computerized dynamic posturography was abnormal in up to 21% of participants. Twenty-two participants had greater than expected white matter changes for age by MRI. Most abnormal findings were dispersed across the population, though a few participants had clusters of abnormalities.

**Conclusions:** Despite our efforts to enroll normal, healthy volunteers, abnormalities on some measures were surprisingly common.

**Trial Registration:** This study was registered at www.clinicaltrials.gov, trial identifier NCT01925963.

## Introduction

The Centers for Disease Control and Prevention report that in 2010, 2.2 million people in the United States sought care at Emergency Departments for traumatic brain injury (TBI) (1). Most TBIs are classified as mild in nature, generally meaning that they result in a relatively brief loss of consciousness (none or <30 min) or interval of altered consciousness or posttraumatic amnesia (<24 h) (2). While most individuals who experience a mild TBI have an uneventful recovery, some have persistent symptoms such as headache, memory complaints, or affective problems (3, 4). A recent prospective study found 22% of individuals with mild TBI experienced functional problems 12 months after injury (5). However, identifying functional deficits in these individuals can be challenging: traditional neuropsychological testing can be insensitive (6), focal neurological findings may be rare or subtle (4, 7), and structural neuroimaging is often normal (8). Assessment of post-concussive symptoms can be sensitive, but these problems occur in other conditions such as chronic pain (9, 10), affective disorders (11), and post-traumatic stress disorder (PTSD) (12). Some providers may interpret the lack of “objective” findings, independent of patient report, as evidence that the patient's complaints are exaggerated.

In addition, the lack of sensitive, widely accepted, validated assessment tools complicates clinical trials of potential treatments for persistent post-concussive symptoms. Some newer assessments do not have robust, well-established norms, while others, such as advanced neuroimaging (13, 14), have inter-equipment and inter-rater variability that limits the utility of published normative data. There is also very little information about how assessments of healthy volunteers across a wide variety of domains correlate with one another.

### Objectives

The U.S. Department of Defense has embarked on a series of trials of hyperbaric oxygen for persistent post-concussive symptoms in military personnel. One of those studies, the *Brain Injury and Mechanisms of Action of hyperbaric oxygen for persistent post-concussive symptoms after mild TBI* (BIMA) study (www.ClinicalTrials.gov: NCT01611194), incorporated extensive outcome measures, including neuroimaging and auditory/vestibular, autonomic, neurological, visual, and sleep function. As a complement to that effort, we conducted an exploratory observational study of healthy volunteers evaluated periodically over 6 months utilizing the same outcome assessments, facilities, equipment, and study personnel. The objective of this study was to develop a normative dataset that could provide information about the distributional properties, expected variability over time, and sensitivity of specific outcome measures in post-concussive symptoms, specifically to inform results from the mild TBI BIMA population (15, 16). We are unaware of any other prospective comprehensive study of those with sequelae following mild TBI who have been compared to volunteers evaluated this extensively and almost identically.

## Materials and Methods

Following institutional review board (IRB) approval from the United States Army Medical Research and Materiel Command IRB (approval number M-10226), volunteers were recruited from the Colorado Springs, Colorado area (elevation 6,000 feet above sea level). Recruitment methods included registration on clinicaltrials.gov (NCT01925963), postings in local establishments or on the internet, radio advertisements, and word of mouth. Interested individuals called a Study Coordinating Center for an initial assessment of eligibility and then were referred to the local site (the Outcomes Assessment Center (OAC), Colorado Springs) for informed consent and in-person assessment.

### Eligibility Criteria

Healthy adult men (18–65 years old) and women (18–35 years old, to match women in the military) without a history of brain injury were eligible for study participation. Participants could be active duty, veteran, or civilian but could not have traveled to a combat zone environment. A history of uncomplicated birth and normal development were required. Participants could not have significant medical or psychological history, nor could they endorse any current brain injury symptoms. Individuals taking daily prescription drugs were excluded except for men at least 45 years old taking statins or angiotensin-converting-enzyme (ACE) inhibitors and women using oral or injectable contraceptives. The full eligibility criteria are listed in Table 1.

**Table 1:** Eligibility criteria.

**INCLUSION CRITERIA**
• Active duty or civilian men and women in the Colorado Springs, Colorado area • Men 18–65 years old and women 18–35 years old at the time of study enrollment • Able to speak and read English, as primary language, and sign the informed consent document • Agrees to and appears able to participate in all outcome assessments, including providing blood samples for laboratory tests and specimen banking
**EXCLUSION CRITERIA**
General exclusions: • Prisoners or minors • Women who are pregnant or breastfeeding • Women of childbearing potential not agreeing to practice an acceptable form of birth control during the study period • Any history of brain injury (trauma, surgery, hypoxia, infection, inflammation, toxicity, or cerebrovascular etiology) • Participation in sports in which a head injury is likely (e.g., mixed martial arts, boxing) during the study period • Concurrent enrollment in any other research trial Significant medical history or condition: • Premature or complicated birth • Developmental delay or learning disorder as a child • Hydrocephalus/microcephaly/macrocephaly • Diabetes mellitus • Atrial septal defect • Known neuroimaging abnormalities • History of therapeutic ionizing radiation to the head • Active malignancy or prior malignancy (except basal cell carcinoma) within the last 5 years Neurological or psychiatric condition or symptoms: • Diagnosis, persistent history, or symptoms of a neurological disorder (e.g., tinnitus, vertigo, chronic fatigue, numbness, tingling, chronic migraine, fibromyalgia, multiple sclerosis) • Active therapy for affective disorders, behavioral disorders, or psychological disorders • Headache that occurs more than twice per week, or migraine or cluster headaches under medical management • Dizziness that occurs more than twice per week or requires medical management • History of theater or war zone activity that placed the participant within a combat zone environment • Diagnosis of post-traumatic stress disorder or sub-clinical post-traumatic symptoms • Current complaints of brain injury symptoms such as cognitive or affective problems (assessed by the OSU TBI-ID) Drug or alcohol abuse history: • Self-reported history of or evidence of illicit drug or marijuana use, except remote (clean for >1 year) non-habitual (greater than weekend) use of marijuana • Self-reported history of alcohol abuse in the past year • Positive urine test for an illicit substance or tetrahydrocannabinol (THC) Daily prescription medication use, except: • Oral or injectable contraceptives • Statins or ACE inhibitors in participants at least 45 years old Confounds or contraindications to the outcome assessments: • Conflicting leave or relocation schedules • Estimated glomerular filtration rate (eGFR) ≥60 • Allergy to iodine-based contrast dye • Anxiety or claustrophobia precluding neuroimaging or vestibular testing • Foreign material in the head or body that would interfere with or pose risk from brain imaging • Unable to abstain from caffeine or tobacco products for at least a 2-h interval • Binocular vision not correctable to 20/50• Deafness in both ears (90 dB HL or greater through the speech frequencies)

### Screening and Enrollment

After obtaining consent, the study team reviewed the participant's self-reported medical history, performed a focused physical examination, and collected a urine specimen to rule out illicit drug use and pregnancy. Traumatic brain injury history was assessed by structured interview (17) and individuals endorsing 1 or more current post-concussive symptoms during this interview were excluded. Potential participants reporting an active mental disorder (receiving current treatment) such as depression, anxiety, and PTSD were excluded. Participants who were asymptomatic at the time of consent but were subsequently found to have underlying pathology were referred for clinical management and, in some cases, withdrawn from the study.

### Outcome Assessments

Participants completed a battery of self-report questionnaires, neuroimaging, autonomic monitoring, sleep assessments, neurological function tests, visual, audiology, and vestibular evaluations, and laboratory tests (Table 2).

**Table 2:** Outcome assessments.

**Assessment domain**	**Baseline**	**13 Weeks**	**6 Months**
**POST-CONCUSSIVE SYMPTOMS AND QUALITY OF LIFE**			
Ohio State University traumatic brain injury identification (OSU TBI-ID)	X	X	X
Neurobehavioral symptom inventory (NSI)	X	X	X
Center for Epidemiological Studies- depression scale (CES- D)	X	X	X
Post-traumatic stress disorder checklist–civilian version (PCL-C)	X	X	X
RAND 36 health survey	X	X	X
World Health Organization quality of life questionnaire (WHOQOL-BREF)	X	X	X
Satisfaction with life scale (SWLS)	X	X	X
**NEUROIMAGING**			
Magnetic resonance imaging (MRI) without gadolinium Arterial spin labeling (ASL) Diffusion tensor imaging (DTI) Proton magnetic resonance spectroscopy (MRS) Functional MRI: resting state, auditory, looming protocol	X X X X X		X X X X X
Computed tomography angiography (CTA) with and without contrast	X		X
**AUTONOMIC FUNCTION**			
24-h Holter monitoring and motion detection	X	X	X
**SLEEP ASSESSMENTS**			
STOP-Bang questionnaire	X		
Restless legs questionnaire	X		
Cataplexy questionnaire	X		
Sleep diary	X		
Actigraphy	X		
Pittsburgh sleep quality index (PSQI)	X	
**NEUROLOGICAL EVALUATION**			
Electroencephalography (EEG)	X		
Brief smell identification test (B-SIT)	X		
6-min walk test (6MWT)	X		
Sharpened Romberg (SRT)	X	X	X
Romberg test	X		
Berg balance scale (BBS)	X		
Neurological examination	X		
Grip strength (dynamometer)	X		
**VISUAL SYSTEM**			
Refractive error	X	X	X
Oculomotor examination	X	X	X
Dynamic visual acuity	X	X	X
Retinal fundoscopy	X	X	X
Dynavision	X	X	X
Eye tracker	X	X	X
**AUDIOLOGY AND VESTIBULAR SYSTEM**			
Vestibular symptoms questionnaire	X	X	X
Peripheral and central auditory examination	X	X	X
Videonystagmography	X	X	X
Computerized dynamic posturography	X	X	X
Rotational vestibular test	X	X	X
VORTEQ active head rotation	X	X	X
Cervical and ocular vestibular evoked myogenic potentials (oVEMP, cVEMP)	X	X	X
**LABORATORY TESTING**			
Illicit drug screening	X	X	X
Pregnancy screening	X	X	X
Comprehensive metabolic panel (CMP)	X	X	X
Glycated hemoglobin (HbA1C)	X		
Complete blood count (CBC) with differential	X		
Flow cytometry	X		
Biological material storage	X		

Self-report questionnaires assessed post-concussive symptoms, depression (18), PTSD (19), and quality of life (20–22). These were administered in paper-and-pencil format. For 24-h ambulatory electrocardiography (ECG), study staff placed a single-channel (lead II) ECG monitor with triaxial accelerometer (Actiwave Cardio, CamNtech, London) on each participant's chest. Data were segmented into wakefulness, sleep, controlled aerobic exercise, and standing still. Linear analysis of cardiac data (NevroKard v.13.2.2, Slovenia) produced heart rate variability measures, including the time between sequential R-waves (R-R intervals), high frequency (parasympathetic) and low frequency (sympathetic) activity, and long-term segment variability.

A board-certified neurologist performed a detailed neurological examination (guided by checklist) assessing mental status, cranial nerves, motor and deep tendon reflexes, gait, cerebellar function, and sensory domains (23). The neurologist evaluated hand grip strength by dynamometer (24) (Tracker Freedom Wireless Grip, JTECH Medical, Midvale, UT, United States) and balance (25) and performed a detailed oculomotor examination, including near point of convergence and the Romberg and Sharpened Romberg tests (23). For the Sharpened Romberg test (26–28), if the participant could not hold their position or changed foot position independent of upper body movement within 30 s, the test was considered positive. Participants attempted four trials, two trials for each foot forward, and the best of the four trials was the score analyzed.

Trained study staff administered the Brief Smell Identification Test (29) and the 6-min walk test, and a certified electroencephalography (EEG) technician performed a 30-min EEG (Cadwell Easy III, Cadwell, Kennewick, WA, United States). The EEG protocol required participants to refrain from caffeine or tobacco for 30 min before the visit and to sleep as normal the night before. The EEG tasks included background rhythm, eyes closed and open, self-reading, basic math problems, hyperventilation, photic stimulation, and a nap opportunity (30). Two board-certified neurologists/clinical neurophysiologists interpreted and scored each EEG, and a third adjudicated in the event of disagreement. The EEG data was also processed using computer algorithms to precisely quantify absolute and relative signal power and the relationships betweens signals recorded at different electrodes (qEEG).

To assess sleep, participants wore an actigraphy device (GTX3, Actigraphy, Pensacola, FL, United States) and completed a sleep diary for a 2-week interval. Participants also completed a series of questionnaires assessing sleep quality and duration (31), risk for sleep apnea (32), restless legs (33), and cataplexy (34, 35).

Participants completed a vestibular symptoms questionnaire (36), and then an AuD audiologist performed a battery of vestibular-balance assessments (36): dynamic visual acuity and posturography (37), rotational chair testing (38), active head rotation (39), videonystagmography (37), and cervical and ocular vestibular evoked potentials (40). The audiologist also performed audiometry and auditory evoked potentials testing (36, 41).

Refractive error (autorefractor) and ocular torsion (retinal fundoscopy) (42, 43) were measured, as were static and dynamic (23, 44) (EDTRS chart) visual acuity. An EyeLink 1000 (SR Research Ltd., Ottawa, ON, Canada) configured for pupil-corneal tracking recorded the horizontal and vertical positions of each eye at 500 Hz as participants performed a series of visual tracking tasks (moving gaze between two static points, horizontal and vertical step and ramp, memory guided, reading, random pursuit, circular, anti-saccade, and horizontal sine) designed in the SR Research Experiment Builder.

Participants received magnetic resonance imaging (MRI) without gadolinium on a 3.0 Tesla scanner (Philips Medical System) with a 32-channel head coil. Images were acquired by 3 certified technologists at maximum spatial resolution while maintaining good signal quality. Anatomical images included T1-weighted (1.0 × 1.0 × 1.0 mm), T2-weighted, T2 FLAIR, and T2^*^-weighted sequences. Quantitative data was collected for mathematical and volumetric analysis of structures. Standard diffusion tensor imaging (DTI) analysis using commercially available FDA-approved software (Olea Sphere; Olea Medical SAS, La Ciotat, France) was performed for fractional anisotropy and mean diffusivity values.

Resting state (i.e., without external stimulation), looming, and auditory functional MRI (fMRI) paradigms were delivered to the patient using the ESys system (InVivo Corporation). In the looming paradigm, two types of visual stimuli (human faces with neutral facial expressions and cars) slowly approached or withdrew from the participant (i.e., expanded or contracted in size) for a 16-s interval. Investigators calculated percent signal change vs. offset of global signal for defined regions of interest in the dorsal interparietal sulcus and ventral premotor. Auditory fMRI tasks included responsive naming, semantic decision, text reading vs. non- linguistic symbols, rhyming, silent word generation, simple object naming, passive listening, visual language comprehension, silent verb generation, word listening, rhyming, and noun-verb semantic association. The fMRI data was analyzed for blood oxygen level dependent (BOLD) tissue enhancement, with resulting brain function activity mapped to the patient's anatomical images.

Participants also underwent water-suppressed multi-voxel proton magnetic resonance spectroscopy (MRS) with point resolved spectroscopy (PRESS) localized above the lateral ventricles and within the brain parenchyma (avoiding calvarial contamination) for N-acetylaspartate, creatinine, and choline.

MRI scans were clinically interpreted by 2 independent neuroradiologists. If there was a discrepancy in the interpretation, the two readers discussed to reach a consensus. If consensus could not be reached, the more conservative of the two interpretations (i.e., the interpretation closer to “normal”) was used. If the participant had significant lesions, those scans were more closely evaluated to determine if there were changes in the lesions over time. Readers were blinded to the order in which they reviewed the scans (baseline and month 6).

Brain perfusion was assessed via two modalities, MRI arterial spin labeling and computed tomography angiography (CTA). Whole brain CTA data was acquired using a 320 × 0.5 mm detector row configuration (Aquilion ONE, Toshiba Medical Systems, Tokyo, Japan), and participants received 50 ml iodinated contrast (Isovue 370, Coviedien Pharmaceutical Products, Hazelwood, Missouri) at 4 ml/sec. DICOM data was reconstructed with Vitria fX software (Vital Images, Minnetonka, MN, United States) using a tracer delay invariant single value decomposition plus deconvolution algorithm. The CT images were clinically interpreted by a single neuroradiologist and were additionally analyzed quantitatively using a combination of independent component analyses and machine learning strategies.

Laboratory testing included comprehensive metabolic panel (CMP), complete blood count (CBC) with differential, human chorionic gonadotropin (female participants of childbearing potential), and carboxyhemoglobin. In addition, participants provided blood for flow cytometry to measure CD34+ and total stem cell count. Serum and plasma was banked for genotyping and future studies. A urine sample was collected for drug screening (all participants) and human chorionic gonadotropin (female participants of childbearing potential).

### Assessment Schedule

**Figure 1:**
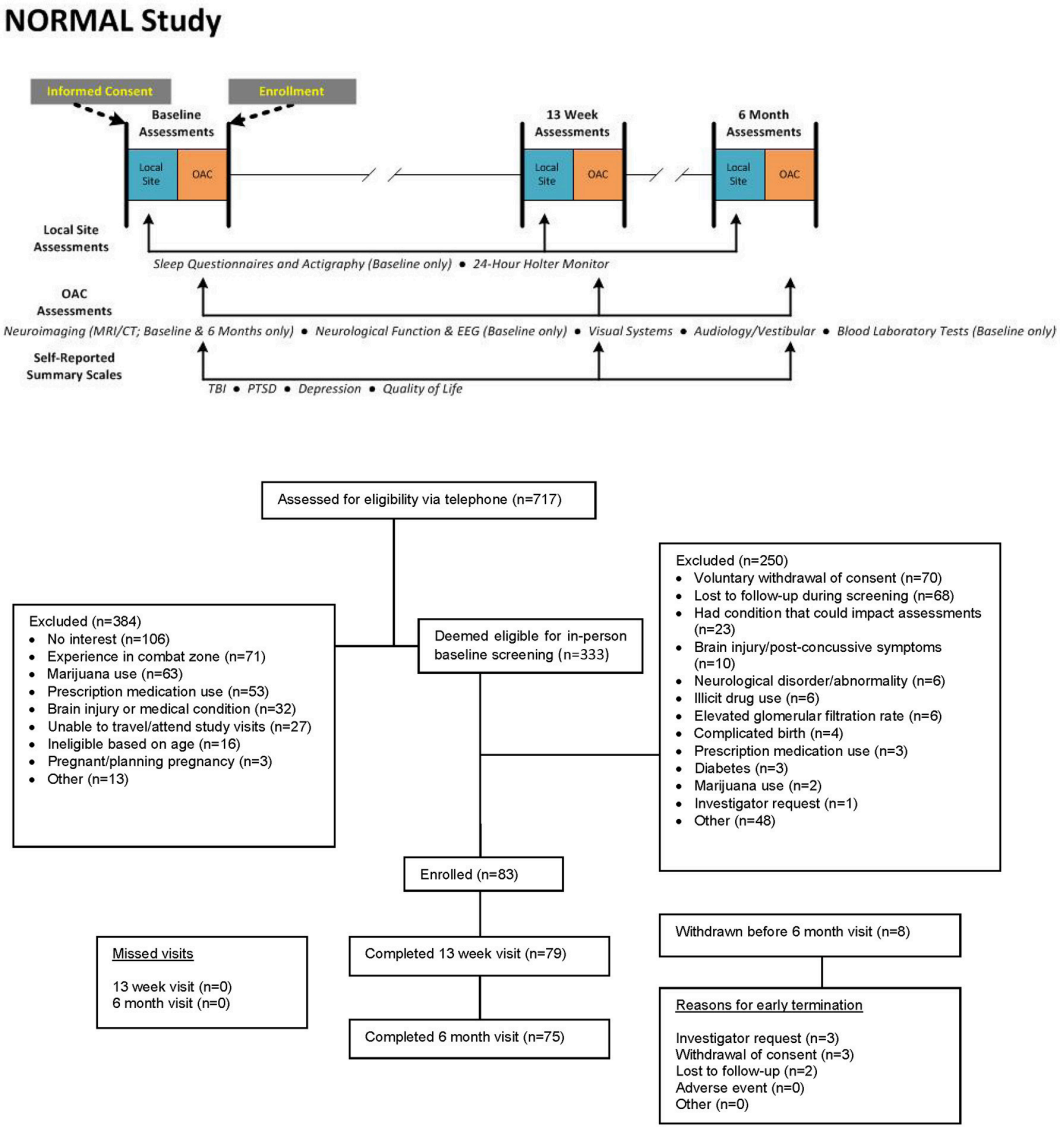
Study design and CONSORT diagram.

The duration of the assessment battery required that the components be scheduled over several days at each testing interval. Participants underwent the complete assessment battery at baseline, at 13 weeks, and 6 months following study enrollment, with the following exceptions (Figure 1):

- Sleep assessments were conducted only at baseline.
- The EEG and comprehensive neurological examination were performed only at baseline. The Sharpened Romberg test was conducted at all three intervals.
- The MRI and CTA were performed at baseline and 6 months.
- Laboratory testing at 13 weeks and 6 months was limited to CMP and drug and pregnancy screening.

The decision to forego some assessments at all three intervals was based upon risk and burden to the participant and allocation of study resources. In addition to the in-person visits, study personnel contacted participants by telephone at 1 and 2 months after enrollment to assess adverse events and maintain communication. Participants were compensated for time and inconvenience as they completed the tests for each interval ($400 for baseline assessments, $600 at 13 weeks, and $800 after completion of the 6-month visit), subject to military and Federal civilian personnel compensation guidelines.

### Statistical Considerations

In this study, neuroimaging data was the primary driver for sample size. Literature on power for quantitative neuroimaging outcomes indicated that a sample size of 10–20 participants per group could provide sufficient statistical power (≥80%) to detect medium to large within-group effect sizes in fMRI activation (45–47), and radiology subject matter experts endorsed 10–15 participants per group as sufficient for radiological interpretation. Therefore, based on age and sex, participants were assigned to 1 of 5 subgroups of up to 15 people: men ages 18–35 years, 36–45 years, 46–55 years, and 56–65 years, and women ages 18–35 years (to approximate the age range of most women in the military), with the intent that age and sex subgroups could be combined for analyses if there were no differences between subgroups. The protocol permitted replacement of participants to fill each subgroup.

Statistical methods were determined a priori. The statistical analysis plan was finalized before data lock, which occurred after the last participant's 6-month assessment. The primary analysis population for this study included all participants who enrolled, completed 13-week and 6-months visits, and were not found to violate inclusion/exclusion criteria following enrollment.

The planned analyses were primarily exploratory in nature and performed with the objective of analyzing the underlying distribution of the outcome assessments and evaluating reliability over time. Univariate tests of change from baseline to each follow-up visit were conducted using paired t-tests for continuous outcomes and McNemar's or exact binomial tests for discrete outcomes. For outcomes measured at follow-up visits, linear mixed models and generalized estimating equations were used to model outcomes over time that showed evidence of change from baseline in univariate testing, adjusting for age and gender subgroups as well as other covariates. Hypothesis testing was two-sided, α = 0.05 level unadjusted for multiple comparisons.

## Results

From January 2014 to January 2016, 717 potential participants were screened by telephone, and 333 were eligible to be screened in-person. Of these, 83 were successfully screened at the site and enrolled in the study, and 75 were included in the primary analysis population (see CONSORT diagram in Figure 1). Baseline characteristics are presented in Table 3. Median age was 38 years (range 18–65 years), 58 (77%) were male, and 69 (92%) had at least some college education. At the time of study enrollment, one was active duty military, 21 (28%) were veterans, and 53 (71%) were civilians.

**Table 3:** Baseline characteristics.

**Characteristic**	**Normal (*n* = 75)**
Age, years, mean (SD)	39.3 (13.3)
Sex, male, n (%)	58 (77.3)
Race, n (%)
Asian	2 (2.7)
American Indian or Alaska Native	1 (1.3)
Black or African American	7 (9.3)
Native Hawaiian or Pacific Islander	1 (1.3)
Other	1 (1.3)
White or Caucasian	62 (82.7)
Not reported	1 (1.3)
Ethnicity, n (%)
Hispanic or Latino	7 (9.3)
Not Hispanic or Latino	67 (89.3)
Not reported	1 (1.3)
Education, n (%)
Less than high school diploma	1 (1.3)
High school diploma	5 (6.7)
Some college	22 (29.3)
College degree	29 (38.7)
Graduate degree	18 (24.0)
Baseline Military Status, n (%)
Active Duty	1 (1.3)
Veteran	21 (28.0)
Civilian	53 (70.7)

Sixty-three participants (84%) reported taking medications or non-prescribed supplements at the baseline assessment interval (median 3, range 1–11); half of the reported drugs were nutritional supplements. Eight women were using oral/continuous contraceptives, and one man used tamsulosin hydrochloride for benign enlarged prostate. Thirty-eight participants reported as-needed use of over-the-counter pain medications, 14 used daily or as-needed decongestants/antihistamines for allergies or upper respiratory illness, 7 used drugs for gastroesophogeal reflux, 3 used daily asthma drugs, and 3 were taking antibiotics or antivirals. Eight participants took aspirin daily for cardiac prophylaxis, 6 took statins, and 4 (all >55 years) used anti-hypertensives.

At baseline, participants did not endorse post-concussive symptoms or symptoms of PTSD or depression (Table 4). Quality of life and life satisfaction scores were at or above average (Table 4). Group mean scores showed little change at 13 weeks and 6 months, though individual participants had some variability as evidenced by wide minimum and maximum change scores (Table 4). Longitudinal models indicated no significant overall effects by time in these outcomes with the exception of WHOQOL-BREF psychological health scores (*p* = 0.04), where a decrease in scores (improvement) was observed over time. *Post-hoc* tests from longitudinal models indicated an estimated mean difference between 6 months and baseline of −2.23 [95% CI (−3.96, −0.50)].

**Table 4:** Standardized symptom and quality of life questionnaire results.

**Measure**	**Baseline score**	**13-week change score**	**6-month change score**
**NEUROBEHAVIORAL SYMPTOM INVENTORY, MEAN (SD) [RANGE]**			
Total score	3.7 (3.5) [0, 15]	0.5 (4.8) [−11, 23]	0.1 (2.8) [−7, 7]
Affective domain	1.8 (1.8) [0, 7]	0.1 (2.6) [−5, 14]	0.1 (1.8) [−5, 5]
Somatic domain	1.2 (1.5) [0, 7]	0.5 (2.2) [−4, 13]	−0.1 (1.1) [−3, 2]
Cognitive domain	0.7 (1.0) [0, 4]	−0.1 (1.1) [−3, 3]	0.0 (1.1) [−3, 3]
**POST-TRAUMATIC STRESS DISORDER CHECKLIST, CIVILIAN VERSION, MEAN (SD) [RANGE]**			
Total score	19.7 (3.5) [17, 34]	0.4 (4.3) [−8, 24]	0.5 (3.8) [−6, 27]
Re-experiencing domain	5.6 (1.3) [5, 12]	0.0 (1.4) [−5, 7]	0.0 (1.5) [−4, 9]
Avoidance/numbing domain	7.9 (1.8) [7, 17]	0.2 (2.2) [−5, 10]	0.2 (1.8) [−4, 11]
Hyperarousal domain	6.1 (1.6) [5, 13]	0.2 (1.7) [−3, 11]	0.3 (1.5) [−3, 7]
**WHOQOL-BREF TRANSFORMED SCORES, MEAN (SD) [RANGE]**			
Physical health	90.7 (8.0) [69, 100]	−1.3 (7.3) [−25, 19]	−1.4 (6.1) [−13, 13]
Psychological	83.0 (11.4) [50, 100]	−1.3 (7.7) [−19, 13]	−2.2 (7.7) [−25, 19]
Social relationships	77.4 (19.9) [6, 100]	−0.3 (15.9) [−50, 50]	−0.6 (13.9) [−37, 44]
Environment	84.9 (13.1) [44, 100]	−1.0 (8.2) [−25, 25]	−1.4 (8.0) [−25, 13]
**CENTER FOR EPIDEMIOLOGIC STUDIES–DEPRESSION SCALE TOTAL SCORE, MEAN (SD) [RANGE]**			
	3.7 (4.2) [0, 25]	0.1 (3.8) [−10, 19]	0.1 (3.9) [−14, 11]
**RAND 36 HEALTH SURVEY TRANSFORMED SCORES, MEAN (SD) [RANGE]**			
Physical functioning	95.1 (16.3) [0, 100]	1.5 (17.1) [−25, 100]	1.9 (16.9) [−25, 100]
Role-physical	99.3 (4.1) [75, 100]	−1.7 (11.9) [−75, 25]	−1.3 (8.1) [−50, 25]
Bodily pain	88.9 (12.7) [42, 100]	−1.9 (13.5) [−38, 22]	−1.1 (15.3) [−59, 30]
General health	86.5 (9.7) [62, 100]	−1.3 (10.6) [−30, 23]	−1.4 (9.2) [−25, 25]
Vitality	71.7 (12.9) [20, 95]	0.0 (11.3) [−30, 25]	−1.3 (10.9) [−40, 20]
Social functioning	96.3 (9.6) [50, 100]	0.2 (11.3) [−37.5, 50]	−0.8 (14.7) [−50, 50]
Role-emotional	93.8 (20.3) [0, 100]	2.7 (24.4) [−66.7, 100]	4.0 (23.2) [−66.7, 100]
Mental health	87.0 (8.6) [52, 100]	0.2 (7.9) [−44, 20]	0.6 (8.7) [−40, 32]
**SATISFACTION WITH LIFE SCALE TOTAL SCORE, MEAN (SD) [RANGE]**			
	28.0 (5.4) [8, 35]	0.4 (3.3) [−9, 11]	0.2 (3.2) [−6, 11]

The neurological examination found infrequent abnormalities: alertness (2 participants), rigidity (1), abnormal jaw reflex (1), heel-to-shin testing (1), and tandem gait (1). All other mental status, cranial nerve, motor, reflex, sensory, and cerebellar testing elements of the neurological examination were normal. All participants had a normal Romberg test, but 16 (21%) could not perform the Sharpened Romberg test to 30 s [compared to expected performance rate of 95% in normal volunteers (28)]. At 13 weeks, 58 (81%) had no change in Sharpened Romberg, 8 (11%) with abnormal Sharpened Romberg at baseline were successful at this interval, and 6 (8%) who could perform this test at baseline could no longer do so. Similar variability was observed at 6 months: 8 previously abnormal participants were successful at 6 months, while 5 who had performed it previously were unsuccessful at this interval.

Thirty-seven of 74 (50%) had near point of convergence >12.7 cm at baseline (48), and this rate in those above 45 years old was 75%. None were rated “impaired” by the Berg Balance Scale. The median number of odors correctly identified on the Brief Smell Identification Test was 11 of 12 (range 6–12). Two participants had abnormal olfactory function relative to age. The median grip strength (both hands) was 66.7 lbs (range 20–112 lbs), and 21 participants had lower-than-expected agerage sustained grip strength (<35 lbs (16 kg) for women and <64 lbs (29 kg) for men). The median distance traveled during the 6-min walk test was 1,816 feet (range 1,226–2,644 feet); only 1 participant walked fewer than 1,312 feet (400 m). Six participants (8%) had generalized slowing on the clinical EEG, but no other EEG abnormalities were noted.

By STOP-Bang questionnaire, one participant was at high risk for obstructive sleep apnea, 13 (17%) were at intermediate risk, and 61 (81%) at low risk. Two participants were symptomatic for restless legs, and no participant reported symptoms of cataplexy. Twenty-one (28%) scored at least 5 on the Pittsburgh Sleep Quality Index global score, indicating poor sleep quality. Median total estimated sleep time was 438 min by sleep diary (99% sleep maintenance efficiency) and 417 min by actigraphy (92% sleep maintenance efficiency). Full sleep results are reported in Table 5.

**Table 5:** Sleep evaluation results.

**Assessment**	**Baseline score**
**PITTSBURGH SLEEP QUALITY INDEX, MEAN (SD) [RANGE]**	
Global score	3.8 (2.2) [1, 11]
Subjective sleep quality component score	0.6 (0.5) [0, 2]
Sleep latency component score	0.8 (0.8) [0, 3]
Sleep duration component score	0.7 (0.6) [0, 2]
Sleep efficiency component score	0.2 (0.6) [0, 3]
Sleep disturbances component score	1.1 (0.3) [1, 2]
Use of sleep medication component score	0.1 (0.5) [0, 3]
Daytime dysfunction component score	0.3 (0.5) [0, 2]
**SLEEP DIARY, MEAN (SD) [RANGE]**	
Total sleep time, minutes	431.2 (45.0) [343, 560]
Wake time after sleep onset, minutes	6.0 (8.5) [0, 45]
Sleep maintenance efficiency, %	96.8 (8.4) [34, 100]
How well did you sleep? (1 = worst, 10 = best)	8.1 (1.1) [5, 10]
**ACTIGRAPHY, MEAN (SD) [RANGE]**	
Total sleep time, minutes	409.3 (53.3) [307, 536]
Wake time after sleep onset, minutes	37.1 (14.4) [5, 49]
Sleep maintenance efficiency, %	91.9 (3.1) [85, 99]

Changes over time, especially at month 6, were identified in several heart rate variability (HRV) outcomes (Table 6), and this finding was particularly evident in analysis of the 24-h segment. In longitudinal models of the 24-h segment (49), significant time effects were identified in log-transformed root square mean of the successive differences (RMSSD) (*p* = 0.01), high frequency (HF) power (*p* = 0.01), log-transformed LF/HF (*p* = 0.01), log-transformed SD1 (*p* = 0.01) and log-transformed SD1/SD2 (*p* = 0.02).

**Table 6:** Heart rate variability measures at baseline and change across time.

Parameter^*^	**Baseline (*n* = 64) Mean (SD) [Range]**	**Change: Baseline to 13 Weeks (*n* = 57)Mean (SD) [95% CI]**	***p*-value**	**Change: Baseline to 6 Months (*n* = 54)Mean (SD) [95% CI]**	***p*-value**
**24-h SEGMENT**					
Standard deviation of RR intervals (SDNN)	177.5 (42.2) [98.2, 287.1]	0.0 (30.7) [−8.18, 8.13]	0.99	−6.1 (37.2) [−16.21, 4.10]	0.24
Root mean square of successive differences of RR intervals (rMSSD)	84.1 (38.7) [26.6, 213.4]	−3.4 (40.3) [−14.07, 7.34]	0.53	−13.8 (49.9) [−27.40, −0.16]	0.047
Ultra low frequency (Normalized units)	967 (1,210) [31, 7,600]	−91 (851) [−317, 135]	0.42	216 (956) [−44, 477]	0.10
Very low frequency (Normalized units)	120.5 (76.5) [29.5, 441.3]	−1.5 (71.5) [−20.50, 17.46]	0.87	16.2 (79.0) [−5.31, 37.80]	0.14
Low frequency power (Normalized units)	59.3 (28.8) [14.9, 124.3]	2.6 (29.4) [−5.20, 10.40]	0.51	8.6 (34.2) [−0.78, 17.91]	0.07
High frequency power (Normalized units)	49.9 (8.6) [32.6, 69.6]	−1.7 (7.5) [−3.64, 0.34]	0.10	−3.1 (7.1) [−5.03, −1.14]	0.002
Very high frequency power (Normalized units)	18.5 (9.8) [2.7, 43.4]	−0.5 (9.6) [−3.01, 2.06]	0.71	−1.3 (13.1) [−4.88, 2.26]	0.46
Low frequency/High frequency (LF/HF) ratio	1.3 (0.8) [0.2, 3.3]	0.2 (0.8) [−0.07, 0.38]	0.16	0.3 (0.9) [0.02, 0.54]	0.03
SD1 (Standard deviation of short axis of ellipse fit to poincare plot)	59.4 (27.4) [18.8, 150.9]	−2.4 (28.5) [−9.95, 5.19]	0.53	−9.7 (35.3) [−19.38, −0.12]	0.05
SD2 (Standard deviation of long axis of ellipse fit to poincare plot)	242.6 (58.4) [134.0, 376.9]	0.9 (40.5) [−9.89, 11.62]	0.87	−6.4 (48.5) [−19.64, 6.83]	0.34
SD1/SD2	0.2 (0.1) [0.1, 0.6]	0.0 (0.1) [−0.04, 0.01]	0.31	0.0 (0.1) [−0.07, −0.01]	0.02
**AWAKE SEGMENT**					
Standard deviation of RR intervals (SDNN)	139.1 (37.7) [61.2, 249.9]	−1.8 (36.4) [−11.43, 7.88]	0.71	−7.8 (41.5) [−19.11, 3.53]	0.17
Root mean square of successive differences of RR intervals (rMSSD)	76.7 (41.6) [21.2, 253.0]	−0.3 (51.9) [−14.11, 13.43]	0.96	−13.5 (58.4) [−29.46, 2.40]	0.09
Ultra low frequency (Normalized units)	514.9 (527.6) [25.4, 3060.6]	−66.5 (484.4) [−195.03, 62.01]	0.30	301.5 (1427.2) [−88.08, 691.02]	0.13
Very low frequency (Normalized units)	113.4 (75.6) [26.6, 489.7]	−0.7 (73.0) [−20.07, 18.68]	0.94	12.3 (73.2) [−7.72, 32.22]	0.22
Low frequency power (Normalized units)	64.3 (32.2) [14.5, 135.1]	2.4 (38.6) [−7.89, 12.59]	0.65	7.1 (39.6) [−3.71, 17.89]	0.19
High frequency power (Normalized units)	42.1 (8.9) [23.0, 63.0]	−1.3 (12.4) [−4.53, 2.03]	0.45	−2.6 (9.1) [−5.07, −0.12]	0.04
Very high frequency power (Normalized units)	21.7 (10.8) [4.7, 50.1]	−0.8 (11.5) [−3.80, 2.28]	0.62	−1.0 (14.3) [−4.93, 2.88]	0.60
Low frequency/High frequency (LF/HF) ratio	1.7 (1.2) [0.4, 5.1]	0.2 (1.6) [−0.20, 0.67]	0.28	0.3 (1.6) [−0.12, 0.75]	0.15
SD1 (Standard deviation of short axis of ellipse fit to poincare plot)	54.2 (29.4) [15.0, 178.9]	−0.2 (36.7) [−9.98, 9.50]	0.96	−9.6 (41.3) [−20.83, 1.70]	0.09
SD2 (Standard deviation of long axis of ellipse fit to poincare plot)	187.5 (50.7) [84.7, 334.8]	−2.2 (45.5) [−14.27, 9.87]	0.72	−8.3 (52.2) [−22.52, 5.97]	0.25
SD1/SD2	0.3 (0.1) [0.1, 0.6]	0.0 (0.2) [−0.05, 0.04]	0.81	0.0 (0.2) [−0.09, 0.0]	0.05
**SLEEP SEGMENT**					
Standard deviation of RR intervals (SDNN)	123.4 (40.5) [46.0, 266.7]	−0.2 (29.1) [−7.93, 7.52]	0.96	−3.3 (33.9) [−12.56, 5.95]	0.48
Root mean square of successive differences of RR intervals (rMSSD)	81.3 (45.2) [16.8, 240.3]	−3.7 (35.3) [−13.01, 5.70]	0.44	−7.6 (39.2) [−18.31, 3.12]	0.16
Ultra low frequency (Normalized units)	266.3 (279.7) [17.6, 1471.3]	−19.5 (307.5) [−101.06, 62.15]	0.63	46.1 (285.9) [−31.92, 124.16]	0.24
Very low frequency (Normalized units)	142.8 (86.5) [23.1, 429.7]	6.2 (100.2) [−20.40, 32.79]	0.64	8.0 (121.1) [−25.04, 41.08]	0.63
Low frequency power (Normalized units)	75.1 (38.8) [15.5, 199.2]	5.4 (41.1) [−5.51, 16.27]	0.33	2.3 (48.3) [−10.90, 15.45]	0.73
High frequency power (Normalized units)	56.4 (12.3) [24.7, 81.7]	−1.2 (9.0) [−3.61, 1.17]	0.31	−1.3 (8.7) [−3.68, 1.05]	0.27
Very high frequency power (Normalized units)	11.4 (8.6) [0.8, 40.2]	−1.1 (8.3) [−3.32, 1.06]	0.31	−0.3 (11.9) [−3.53, 2.95]	0.86
Low frequency/high frequency (LF/HF) ratio	1.5 (1.3) [0.2, 7.2]	0.1 (1.5) [−0.28, 0.52]	0.54	0.0 (1.4) [−0.38, 0.36]	0.97
SD1 (Standard deviation of short axis of ellipse fit to poincare plot)	57.5 (32.0) [11.9, 169.9]	−2.6 (24.9) [−9.20, 4.03]	0.44	−5.4 (27.8) [−12.94, 2.21]	0.16
SD2 (Standard deviation of long axis of ellipse fit to poincare plot)	163.7 (51.2) [64.0, 336.7]	0.9 (36.6) [−8.85, 10.59]	0.86	−2.8 (43.0) [−14.57, 8.89]	0.63
SD1/SD2	0.3 (0.1) [0.2, 0.9]	0.0 (0.1) [−0.04, 0.02]	0.44	0.0 (0.1) [−0.07, 0.01]	0.09
**EXERCISE SEGMENT**					
Standard deviation of RR intervals (SDNN)	136.8 (36.2) [77.4, 229.8]	−0.4 (31.8) [−8.85, 8.04]	0.92	−9.2 (31.8) [−17.84, −0.49]	0.04
Root mean square of successive differences of RR intervals (rMSSD)	54.7 (36.0) [13.6, 166.9]	0.7 (44.4) [−11.08, 12.49]	0.90	−7.7 (43.6) [−19.59, 4.24]	0.20
Ultra low frequency (Normalized units)	1873 (1,643) [91, 8,048]	−224 (1,763) [−692, 243]	0.34	69 (2,351.0) [−573, 711]	0.83
Very low frequency (Normalized units)	135.5 (98.0) [24.9, 575.4]	−8.3 (98.7) [−34.5, 17.9]	0.53	21.0 (97.0) [−5.5, 47.5]	0.12
Low frequency power (Normalized units)	74.3 (47.7) [13.7, 227.6]	−5.9 (54.2) [−20.26, 8.53]	0.42	6.1 (48.1) [−7.00, 19.25]	0.35
High frequency power (Normalized units)	35.4 (10.0) [16.1, 59.5]	1.8 (12.0) [−1.40, 4.95]	0.27	−0.9 (10.1) [−3.67, 1.84]	0.51
Very high frequency power (Normalized units)	22.8 (13.5) [1.9, 54.5]	0.0 (16.6) [−4.39, 4.44]	0.99	−1.1 (15.9) [−5.45, 3.25]	0.61
Low frequency/high frequency (LF/HF) ratio	2.7 (2.6) [0.3, 13.2]	−0.4 (2.9) [−1.14, 0.42]	0.36	0.2 (2.4) [−0.46, 0.83]	0.57
SD1 (Standard deviation of short axis of ellipse fit to poincare plot)	38.7 (25.5) [9.6, 118.0]	0.5 (31.4) [−7.84, 8.83]	0.90	−5.4 (30.9) [−13.85, 3.00]	0.20
SD2 (Standard deviation of long axis of ellipse fit to poincare plot)	188.3 (49.7) [106.5, 305.6]	−0.7 (41.6) [−11.73, 10.37]	0.90	−11.8 (42.1) [−23.30, −0.34]	0.04
SD1/SD2	0.2 (0.1) [0.1, 0.6]	0.0 (0.2) [−0.04, 0.04]	0.95	0.0 (0.2) [−0.06, 0.03]	0.48

* *Not all parameters are appropriate for reporting all segments, but all data values are included here for the sake of completeness*.

No significant overall time effects were identified for HRV outcomes in the sleep segment, suggesting greater stability of outcomes during this period of the ECG recording. Although some differences in HRV outcomes were expected at baseline between age and gender groups, differences between the subgroups in changes over a 6 month time period were not necessarily expected. Differences in changes over time between age and gender groups were observed in outcomes in several segments, most notably the 24-h segment. Results of longitudinal models indicated that no significant overall age and gender-by-time interactions were observed in outcomes from the sleep segment, suggesting that HRV outcomes measured during sleep may be the least susceptible to noise and best for future studies.

In the visual system evaluation (Table 6), no participant experienced a myopic change >1 spherical equivalent as measured by autorefractor over the course of the study. With both eyes open, all participants had normal dynamic visual acuity (by EDTRS chart) at baseline, but 1 participant was abnormal at 13 weeks and 6 months. Forty-one of 72 participants (57%) had a fundus angle >7°, and 21 (29%) had a significant change in fundus angle (normal to abnormal, or abnormal to normal) at 13 weeks compared to baseline. All participants performed within the normal range on the Dynavision reaction time, self-paced, and forced attention tests. Changes in visual, motor, and physical reaction time were not significant over time, but participants were able to perform significantly more self-paced and forced attention hits at 13 weeks and 6 months.

By eye tracker, participants were most likely to have abnormalities on the circular, horizontal ramp, and reading tasks (Table 7). Forty participants (53%) had normal performance on all 3 tasks at all 3 timepoints. Another 16 participants (21%) were abnormal on just 1 task at any timepoint. Thirteen participants had 2 or 3 abnormal scores, and 6 participants had 4 or more abnormal scores.

**Table 7:** Visual system evaluation results.

	**Baseline**	**13 Weeks**			**6 Months**		
		**Nochange**	**Normal to abnormal**	**Abnormal to normal**	**Nochange**	**Normal to abnormal**	**Abnormal tonormal**
**EDTRS DYNAMIC VISUAL ACUITY, AT LEAST 10 LINES LOST, N (%)**							
Right eye	2 (2.7)	72 (96)	1 (1)	2 (3)	72 (96)	2 (3)	1 (1)
Left eye	0 (0)	74 (99)	1 (1)	0 (0)	75 (100)	0 (0)	0 (0)
Both eyes	0 (0)	74 (99)	1 (1)	0 (0)	74 (99)	1 (1)	0 (0)
**FUNDUS ANGLE** **>7°, N (%)**							
	41 (57)	50 (70)	8 (11)	13 (18)	58 (82)	5 (7)	8 (11)
**DYNAVISION, N (%) ABNORMAL**							
Self-paced hits ≤51	0 (0)	75 (100)	0 (0)	0 (0)	75 (100)	0 (0)	0 (0)
60 s forced attention hits ≤51	0 (0)	75 (100)	0 (0)	0 (0)	75 (100)	0 (0)	0 (0)
	**BaselineMean (SD)[Range]**		**13-weekchange scoreMean (SD) [95% CI]**		***p*****-value**	**6-monthchange scoreMean (SD) [95% CI]**	***p*****-value**
**DYNAVISION**									
Reaction time test^*^					
Visual reaction time, sec	0.34 (0.05) [0.26, 0.60]		0.00 (0.04) [−0.01, 0.01]		0.48	−0.01 (0.04) [−0.02, 0.0]	0.12
Motor reaction time, sec	0.22 (0.07) [0.11, 0.42]		0.00 (0.06) [−0.19, 0.09]		0.91	0.00 (0.07) [−0.02, 0.01]	0.54
Physical reaction time, sec	0.56 (0.10) [0.40, 1.01]		0.00 (0.07) [−0.02, 0.01]		0.80	−0.01 (0.09) [−0.03, 0.01]	0.19
Self-paced hits^**^	77.7 (7.2) [59, 102]		1.6 (6.8) [0.02, 3.15]		0.047	3.8 (7.1) [2.15, 5.42]	<0.001
60-second forced attention hits^**^	70.3 (7.7) [52, 88]		1.3 (6.6) [−0.22, 2.84]		0.09	3.6 (6.6) [2.06, 5.11]	<0.001
	**Baseline**			**13 weeks**			**6 months**		
**EYE TRACKER**								
Abnormal circular task, n	11			3			8		
Abnormal horizontal ramp task, n	5			5			10		
Abnormal reading task, n	14			8			2		

* *Averaged across right and left hand*.

** *Values summed across all quadrants*.

Clinical interpretation of vestibular and audiology test results are presented in Table 8. During administration of the Vestibular Symptoms Questionnaire at baseline, 12 participants (16%) reported some hearing loss and 11 (15%) reported tinnitus. Ten (13%) reported provocation of vestibular symptoms during motion activities in the direct vestibular assessment. Baseline videonystagmography was normal for most participants. Four (5%) had abnormal head thrust and head shake, and 22 (30%) had an abnormal response to monothermal, warm air caloric testing. On computerized dynamic posturography, sensory organization testing was normal; however, during the dynamic visual acuity component, 10–21% of participants had abnormal test results, depending on the parameter measured. During the rotational vestibular test, no participant had nystagmus and 4 (5%) had square wave jerks. Abnormal vertical saccades were more common than horizontal and most frequently seen in the velocity domain. Ten participants (13%) were unable to even partially complete the VORTEQ head velocity test under the 4 kHz horizontal test condition, and 1 failed the 3 kHz vertical test. Ocular VEMPs were absent in 32 participants (43%) at baseline. While this finding is difficult to interpret in isolation, participants reported that ocular VEMPs were fatiguing, and some failed to maintain an upward gaze, which resulted in invalid testing.

**Table 8:** Vestibular and audiology system results.

	**Baseline**	**13 Weeks**			**6 Months**		
		**No change**	**Normal to abnormal**	**Abnormal to normal**	**No change**	**Normal to abnormal**	**Abnormal to normal**
**VESTIBULAR SYMPTOMS QUESTIONNAIRE, N (%) ABNORMAL**							
Vestibular deficit^*^	13 (17)	61 (81)	5 (7)	9 (12)	65 (87)	2 (3)	8 (11)
**Direct Vestibular Assessment**							
Vestibulo-ocular reflex abnormalities
Quick turn of head	0 (0)	74 (99)	1 (1)	0 (0)	75 (100)	0 (0)	0 (0)
Walking	0 (0)	74 (99)	1 (1)	0 (0)	75 (100)	0 (0)	0 (0)
Moving objects in visual field	1 (1)	73 (97)	1 (1)	1 (1)	74 (99)	0 (0)	1 (1)
Walking at night in poor visibility	4 (5)	74 (99)	1 (1)	0 (0)	72 (96)	0 (0)	3 (4)
Tolerance to motion activities	10 (13)	68 (91)	1 (1)	6 (8)	69 (92)	1 (1)	5 (7)
**Dizziness/vestibular systems**							
Spinning	0 (0)	75 (100)	0 (0)	0 (0)	75 (100)	0 (0)	0 (0)
Lightheadedness	3 (4)	71 (95)	1 (1)	3 (4)	70 (93)	2 (3)	3 (4)
Instability/drunk-like feeling	0 (0)	74 (99)	1 (1)	0 (0)	75 (100)	0 (0)	0 (0)
**Non-direct vestibular symptoms**							
Hearing loss	12 (16)	65 (87)	4 (5)	6 (8)	65 (87)	2 (3)	8 (11)
Tinnitus	11 (15)	71 (95)	3 (4)	1 (1)	70 (93)	2 (3)	3 (4)
Headaches	5 (7)	67 (89)	4 (5)	4 (5)	66 (88)	5 (7)	4 (5)
Facial Numbness	0 (0)	74 (99)	1 (1)	0 (0)	75 (100)	0 (0)	0 (0)
Anxiety	5 (7)	69 (92)	1 (1)	5 (7)	65 (87)	5 (7)	5 (7)
Change in vision	2 (3)	73 (97)	0 (0)	2 (3)	73 (97)	0 (0)	2 (3)
Pain	16 (21)	59 (79)	5 (7)	11 (15)	52 (69)	9 (12)	14 (19)
Syncope	0 (0)	75 (100)	0 (0)	0 (0)	75 (100)	0 (0)	0 (0)
**VIDEONYSTAGMOGRAPHY, N (%) ABNORMAL**							
Conjugate eye movement	0 (0)	75 (100)	0 (0)	0 (0)	75 (100)	0 (0)	0 (0)
Head thrust	4 (5)	69 (92)	2 (3)	4 (5)	69 (92)	2 (3)	4 (5)
Spontaneous nystagmus	0 (0)	75 (100)	0 (0)	0 (0)	74 (99)	1 (1)	0 (0)
Pneumotoscopy	0 (0)	75 (100)	0 (0)	0 (0)	75 (100)	0 (0)	0 (0)
Nasal pinch valsalva	0 (0)	75 (100)	0 (0)	0 (0)	75 (100)	0 (0)	0 (0)
Glottal pressure	0 (0)	75 (100)	0 (0)	0 (0)	75 (100)	0 (0)	0 (0)
Head shake	4 (5)	69 (93)	2 (3)	3 (4)	68 (92)	3 (4)	3 (4)
Dix-Hallpike	0 (0)	75 (100)	0 (0)	0 (0)	75 (100)	0 (0)	0 (0)
Calorics (mono-thermal, warm air)	22 (30)	45 (63)	15 (21)	11 (16)	49 (72)	9 (13)	10 (15)
**DYNAMIC VISUAL ACUITY, N (%) ABNORMAL**							
Horizontal	7 (10)	48 (79)	7 (12)	6 (10)	50 (81)	7 (11)	5 (8)
Vertical	12 (17)	38 (62)	13 (21)	10 (16)	48 (77)	6 (10)	8 (13)
Roll	15 (21)	39 (65)	14 (23)	7 (12)	35 (57)	13 (21)	13 (21)
**SENSORY ORGANIZATION TEST, N (%) ABNORMAL**							
Condition 1	0 (0)	75 (100)	0 (0)	0 (0)	74 (99)	1 (1)	0 (0)
Condition 2	0 (0)	74 (99)	1 (1)	0 (0)	75 (100)	0 (0)	0 (0)
Condition 3	1 (1)	75 (100)	0 (0)	0 (0)	74 (99)	0 (0)	1 (1)
Condition 4	1 (1)	74 (99)	0 (0)	1 (1)	74 (99)	0 (0)	1 (1)
Condition 5	1 (1)	74 (99)	0 (0)	1 (1)	74 (99)	0 (0)	1 (1)
Condition 6	0 (0)	74 (99)	1 (1)	0 (0)	75 (100)	0 (0)	0 (0)
**ROTATIONAL VESTIBULAR TEST, N (%) ABNORMAL**							
Pre-assessment spontaneous nystagmus	1 (1)	73 (97)	1 (1)	1 (1)	74 (99)	0 (0)	1 (1)
Post-assessment spontaneous nystagmus	71 (96)	70 (97)	1 (1)	1 (1)	70 (94)	3 (4)	1 (1)
Nystagmus	0 (0)	75 (100)	0 (0)	0 (0)	75 (100)	0 (0)	0 (0)
Square wave jerks	4 (5)	68 (91)	4 (5)	3 (4)	69 (92)	3 (4)	3 (4)
**Horizontal saccades**						
Tracing characteristics	0 (0)	74 (100)	0 (0)	0 (0)	74 (100)	0 (0)	0 (0)
Accuracy	8 (11)	61 (82)	7 (10)	6 (8)	68 (92)	2 (3)	4 (5)
Velocity	9 (12)	60 (81)	11 (15)	3 (4)	50 (68)	17 (23)	7 (10)
Latency	3 (4)	66 (89)	5 (7)	3 (4)	63 (85)	8 (11)	3 (4)
**Vertical saccades**						
Tracing characteristics	3 (4)	71 (96)	0 (0)	3 (4)	67 (94)	1 (1)	3 (4)
Accuracy	18 (24)	50 (68)	12 (16)	12 (16)	53 (75)	7 (10)	11 (16)
Velocity	29 (39)	51 (69)	11 (15)	12 (16)	48 (68)	15 (21)	8 (11)
Latency	4 (5)	68 (92)	3 (4)	3 (4)	66 (93)	4 (6)	1 (1)
Static subjective visual vertical	19 (25)	56 (75)	6 (8)	13 (17)	57 (76)	5 (7)	13 (17)
Static subjective visual horizontal	11 (15)	60 (81)	7 (10)	7 (10)	62 (84)	5 (7)	7 (10)
Oculomotor smooth pursuit	5 (7)	70 (93)	4 (5)	1 (1)	67 (89)	6 (8)	2 (3)
**VORTEQ HEAD VELOCITY TEST**							
4 khz horizontal, n (%) failure	10 (13)	68 (91)	2 (3)	5 (7)	66 (88)	2 (3)	7 (9)
3 khz vertical, n (%) failure	1 (1)	73 (97)	1 (1)	1 (1)	72 (96)	2 (3)	1 (1)
**VESTIBULAR EVOKED POTENTIALS**							
Cervical VEMPs 95D bnHL response, n (%) absent	6 (8)	64 (87)	6 (8)	4 (5)	65 (89)	4 (6)	4 (6)
Ocular VEMPs 95D bnHL Response, n (%) absent	32 (43)	47 (64)	12 (16)	15 (20)	53 (73)	11 (15)	9 (12)
**AUDITORY TESTING, N (%) ABNORMAL (>25 dBHL)**							
Quick Speech in Noise (QuickSIN) score >3	1 (1)	70 (100)	0 (0)	0 (0)	69 (99)	0 (0)	1 (1)
Hearing loss–speech reception thresholds	3 (4)	73 (97)	0 (0)	2 (3)	73 (97)	0 (0)	2 (3)
Hearing loss–pure tone averages	5 (7)	74 (99)	0 (0)	1 (1)	73 (97)	0 (0)	2 (3)
Pure tone air conduction thresholds −4 kHz	16 (21)	72 (96)	0 (0)	3 (4)	72 (96)	0 (0)	3 (4)
Pure tone air conduction thresholds −8 kHz	14 (19)	73 (97)	1 (1)	1 (1)	75 (100)	0 (0)	0 (0)
Reliability of speech reception thresholds and pure tone averages^**^	10 (13)	65 (87)	2 (3)	8 (11)	63 (84)	4 (5)	8 (11)
**PERIPHERAL AUDITORY ASSESSMENT, N (%) ABNORMAL**							
Transient otoacoustic emissions	17 (23)	68 (91)	1 (1)	6 (8)	71 (95)	1 (1)	3 (4)
Functional otoscopy	2 (3)	69 (95)	2 (3)	2 (3)	71 (97)	0 (0)	2 (3)
Middle ear tympanometry	15 (20)	63 (85)	2 (3)	9 (12)	58 (80)	6 (8)	9 (12)
**CENTRAL AUDITORY ASSESSMENT, N (%) ABNORMAL**							
SCAN3:A	8 (16)	43 (88)	1 (2)	5 (10)	40 (82)	3 (6)	6 (12)
Auditory late response	2 (3)	67 (91)	5 (7)	2 (3)	66 (90)	5 (7)	2 (3)
Auditory brainstem response	3 (4)	73 (97)	0 (0)	2 (3)	71 (95)	1 (1)	3 (4)
Auditory brainstem response stress	18 (24)	48 (64)	15 (20)	12 (16)	50 (68)	12 (16)	12 (16)
Middle latency response	24 (32)	44 (59)	20 (27)	11 (15)	46 (61)	17 (23)	12 (16)
Auditory steady-state response	22 (29)	57 (76)	8 (11)	10 (13)	55 (73)	9 (12)	11 (15)

* *Vestibular deficit defined as: (1) feeling dizzy/imbalanced while walking at night in poor visibility; (2) mild or worse abnormalities in other vestibulo-ocular reflex symptoms; or (3) dizziness/vestibular symptoms from direct vestibular assessment*.

** *Poor reliability ≥10 dB difference between speech reception thresholds and pure tone averages for either ear*.

On auditory testing, few participants had hearing loss defined as >25 dbHL (3 by speech reception thresholds and 5 by pure tone averages). Reliability of speech reception thresholds and pure tone averages was 87% (<10 dB difference between the two measurements). Twenty to 30% had abnormal features of their peripheral and central auditory assessments. Most vestibular and audiology measures were stable over time. Although at least 20% of participants had significant interval-to-interval changes in pain reporting, dynamic visual acuity performance, some horizontal and vertical saccades domains, subjective visual vertical, ocular VEMPs, and some central auditory measures, longitudinal models indicated no significant overall time effects in these assessments.

Neuroimaging abnormalities were surprisingly common in this population that was carefully selected to be healthy, without prior brain injury. The clinical MRI interpretation was positive at baseline in 45 participants (61%) for non-specific white matter changes (e.g., T2 white matter hyperintensities). Other common findings were diffusion tensor imaging (44, 60%), cavum septum (32, 46%), dilated perivascular spaces (34, 47%), and pineal cysts (31, 44%). Based on overall clinical impression of the individual scans, only 34 participants (45%) had no white matter lesions, while 22 were identified by the neuroradiologists as having a lesion burden (based on number and size of lesions) greater than expected for age. The remaining 19 participants had white matter lesions but the number and size may be within the expected range for age (50, 51). When comparing baseline and 6-month scans individually, the apparent lesion burden increased in 19 (26%) and decreased in 5 (7%) (*p* = 0.07), but when these scans were compared side-by-side, the neuroradiologists found 96% of participants had no significant changes in their MRI, and the observable changes were in mastoid fluid and sinus disease, which were common at baseline in this population (38, 54%).

With regard to quantitative analysis (by FreeSurfer and Neuroquant), significant increases from baseline to month 6 were observed in several regions of interest, primarily in white matter volumes (data not shown). However, some statistically significant changes were expected given the large number of regions measured. Although some baseline differences were observed between age and gender groups in FreeSurfer outcomes, no significant age-by-time or gender-by-time interactions were observed, suggesting stability over time across subgroups.

On diffusion tensor imaging, the mean axial diffusivity across the corpus callosum was 1.58 ± 0.06 (range 1.38–1.71) and the radial diffusivity was 0.51 ± 0.03 (range 0.43–0.58) at baseline. No clinically significant changes were observed over time. Two participants had both fractional anisotropy and radial diffusivity measures that were >2 standard deviations outside the mean.

Relative metabolite ratios for MR spectroscopy are listed in Table 9. Auditory and resting state fMRI data will be presented elsewhere. On the looming measure, the study population as a whole had significantly decreased responses from baseline to month 6 to face stimuli, specifically in the right hemispheres of the dorsal interparietal sulcus and ventral premotor areas.

**Table 9:** Neuroimaging findings.

	**Baseline**		**6 Months**		
	**Complete data**	**Abnormal, n (%)**	**No change**	**Normal to abnormal**	**Abnormal to normal**
**CLINICAL MRI INTERPRETATION**					
Aneurysm	70	0 (0)	67 (100)	0 (0)	0 (0)
Arachnoid cysts	70	3 (4)	66 (99)	1 (1)	0 (0)
Arterial anatomical variations	70	0 (0)	67 (100)	0 (0)	0 (0)
Asymmetrical ventricles	70	8 (11)	63 (94)	2 (3)	2 (3)
Brain atrophy	71	10 (14)	60 (88)	4 (6)	4 (6)
Cavum septum	70	32 (46)	52 (75)	4 (6)	13 (19)
Contusions	70	0 (0)	67 (100)	0 (0)	0 (0)
Diffuse or traumatic axonal injuries (T2 hyperintensities)	74	45 (60)	64 (89)	7 (10)	1 (1)
Diffusion tensor imaging	73	44 (60)	54 (75)	11 (15)	7 (10)
Developmental venous abnormalities	71	4 (6)	64 (94)	0 (0)	4 (6)
Encephalomalacia	70	2 (3)	66 (99)	1 (2)	0 (0)
Gliosis	70	1 (1)	66 (99)	0 (0)	1 (2)
Intracerebral hemorrhages	70	1 (1)	66 (99)	0 (0)	1 (2)
Lymph nodes	71	7 (10)	61 (90)	2 (3)	5 (7)
Mastoid fluid	70	1 (1)	65 (97)	1 (2)	1 (2)
Other	70	9 (13)	61 (90)	4 (6)	3 (4)
Pineal cysts	70	31 (44)	56 (84)	3 (5)	8 (12)
Pituitary abnormalities	71	13 (18)	62 (91)	1 (2)	5 (7)
Dilated perivascular spaces	72	34 (47)	45 (64)	3 (4)	22 (31)
Sinus disease	71	38 (54)	47 (68)	8 (12)	14 (20)
Venous anatomical variations	70	2 (3)	65 (97)	1 (2)	1 (2)
Ventricular enlargement	70	1 (1)	65 (97)	2 (3)	0 (0)
Venous sinus injury	70	0 (0)	67 (100)	0 (0)	0 (0)
**DIFFUSION TENSOR IMAGING**					
Corpus callosum: genu	73	2 (3)	69 (96)	1 (1)	2 (3)
Corpus callosum: anterior body	73	25 (34)	48 (67)	16 (22)	8 (11)
Corpus callosum: mid body	73	8 (11)	65 (90)	2 (3)	5 (7)
Corpus callosum: posterior body	73	42 (58)	51 (71)	11 (15)	10 (14)
Corpus callosum: splenium	73	2 (3)	69 (96)	2 (3)	1 (1)
**CLINICAL CT INTERPRETATION**					
Aneurysm	75	1 (1)	74 (100)	0 (0)	0 (0)
Arachnoid cysts	75	6 (8)	74 (100)	0 (0)	0 (0)
Arterial anatomical variations	75	41 (55)	72 (97)	2 (3)	0 (0)
Asymmetrical ventricles	75	3 (4)	74 (100)	0 (0)	0 (0)
Cavum septum	75	1 (1)	74 (100)	0 (0)	0 (0)
Intracerebral hemorrhages	75	0 (0)	74 (100)	0 (0)	0 (0)
Mastoid fluid	75	0 (0)	74 (100)	0 (0)	0 (0)
Microhemorrhages	75	0 (0)	74 (100)	0 (0)	0 (0)
Other	75	5 (7)	74 (100)	0 (0)	0 (0)
Pineal cysts	75	0 (0)	74 (100)	0 (0)	0 (0)
Sinus disease	75	6 (8)	73 (99)	0 (0)	1 (1)
Venous anatomical variations	75	8 (11)	72 (97)	0 (0)	2 (7)
Ventricular enlargement	75	3 (4)	74 (100)	0 (0)	0 (0)
Venous sinus injury	75	1 (1)	73 (99)	0 (0)	1 (1)
**CT PERFUSION INFORMATION**					
Cerebral blood flow	75	16 (21)	69 (93)	2 (3)	3 (4)
Cerebral blood volume	75	17 (23)	69 (93)	2 (3)	3 (4)
Functional delay	75	12 (16)	69 (93)	3 (4)	2 (3)
Mean transit time	75	17 (23)	69 (93)	2 (3)	3 (4)
Time-to-peak	75	16 (21)	69 (93)	2 (3)	3 (4)
Volumetric surface	75	0 (0)	73 (99)	1 (1)	0 (0)
**Quantitative diffusion tensor imaging (n= 74) Mean (SD) [Range]**	**Mean fractional anisotropy**		**Mean fractional diffusivity**	**Mean axial diffusivity**	**Mean radial diffusivity**
Corpus Callosum Total	0.62 (0.02) [0.59, 0.67]		0.86 (0.03) [0.76, 0.94]	1.58 (0.06) [1.38, 1.71]	0.51 (0.03) [0.43, 0.58]
Corpus Callosum Anterior Inferior	0.54 (0.03) [0.47, 0.61]		0.86 (0.05) [0.72, 0.97]	1.46 (0.07) [1.29, 1.61]	0.56 (0.05) [0.44, 0.67]
Corpus Callosum Anterior	0.56 (0.03) [0.50, 0.63]		0.98 (0.08) [0.78, 1.23]	1.67 (0.12) [1.39, 2.03]	0.64 (0.06) [0.47, 0.83]
Corpus Callosum Mid-body	0.59 (0.07) [0.36, 0.73]		1.11 (0.18) [0.84, 1.80]	1.89 (0.18) [1.50, 2.59]	0.72 (0.19) [0.44, 1.41]
Corpus Callosum Posterior Inferior	0.69 (0.02) [0.64, 0.75]		0.79 (0.04) [0.68, 0.92]	1.56 (0.08) [1.32, 1.75]	0.41 (0.03) [0.33, 0.52]
Corpus Callosum Posterior	0.68 (0.04) [0.52, 0.77]		0.98 (0.11) [0.74, 1.27]	1.83 (0.15) [1.40, 2.17]	0.55 (0.11) [0.38, 0.89]
Corpus Callosum Genu	0.55 (0.03) [0.48, 0.60]		0.89 (0.05) [0.74, 1.00]	1.51 (0.07) [1.33, 1.67]	0.58 (0.04) [0.45, 0.69]
Corpus Callosum Splenium	0.69 (0.02) [0.64, 0.74]		0.82 (0.04) [0.70, 0.93]	1.60 (0.08) [1.35, 1.78]	0.43 (0.03) [0.35, 0.53]
**Baseline quantitative MR spectroscopy metabolite ratios Mean (SD)**	**Left**			**Right**	
	**Baseline**		**6 Months**	**Baseline**	**6 Months**
N-acetylaspartate/Creatine	2.10 (0.22)		2.07 (0.20)	1.98 (0.20)	1.95 (0.22)
Choline/Creatine	0.96 (0.12)		0.94 (0.11)	0.88 (0.10)	0.87 (0.10)
Choline/ N-acetylaspartate	0.46 (0.07)		0.46 (0.07)	0.45 (0.06)	0.45 (0.07)

Images acquired via arterial spin labeling were of poor quality and contained no useable information about brain perfusion. Clinical interpretation of CTA was more sensitive than that of MRI in identifying vascular anatomical variations (Table 9), but less sensitive in identification of other structural abnormalities. While the volumetric surfaces were normal, other perfusion measures were abnormal in 16–23% of participants. Perfusion tended to be stable over time (Table 9).

All participants had CD34+ and total stem cell counts within the normal range (mean 0.04 ± 0.01% and 2.1 ± 1.0 cells/uL, respectively).

### Population Distribution of Abnormalities

**Figure 2:**
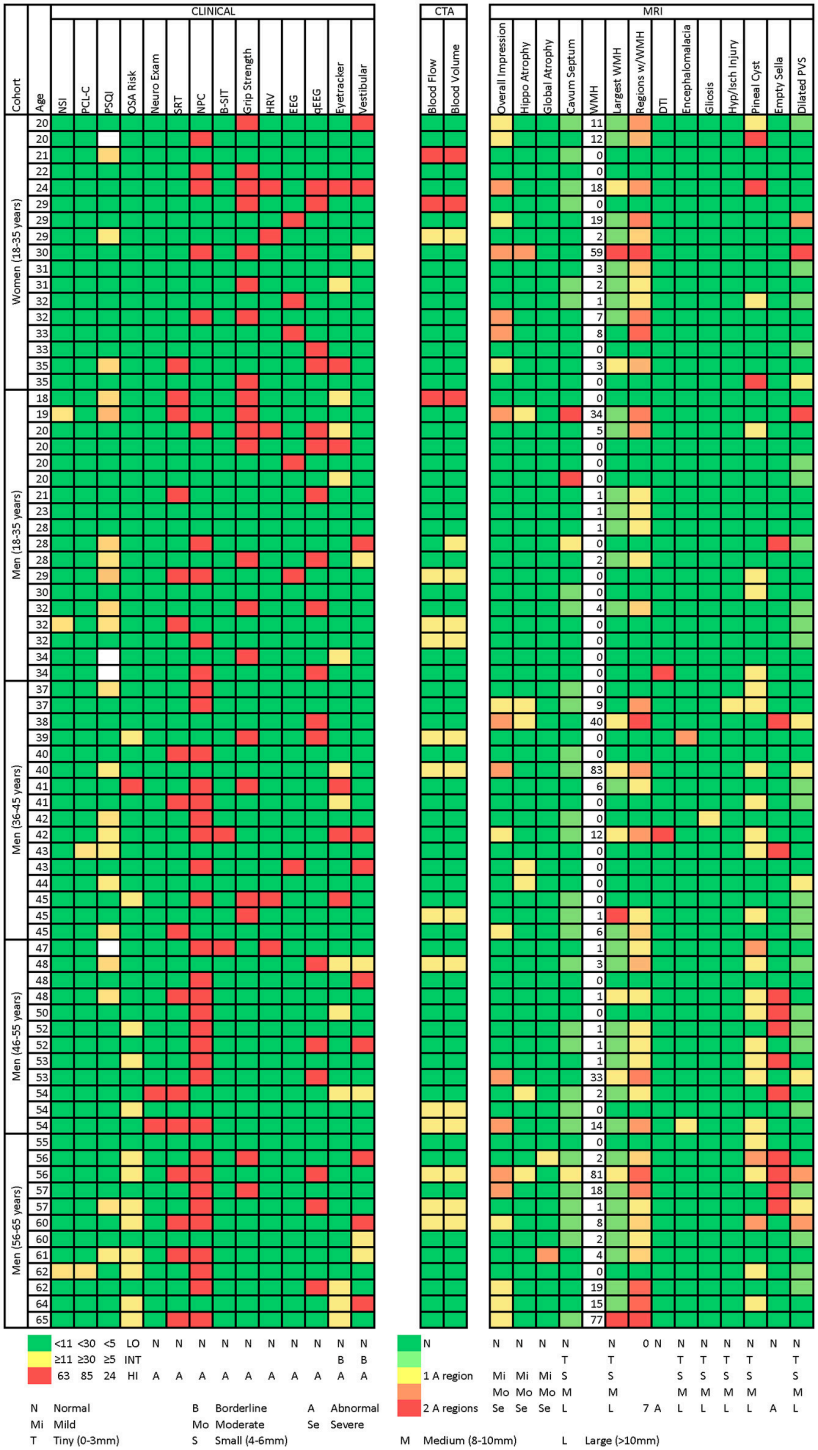
Heatmap of abnormalities over selected measures for age and gender subgroups. The following methods were used to generate this figure: 1. Neurobehavioral Symptom Inventory (NSI) total score: green if ≤10, yellow to red from 11 to 63 (maximum possible score). 2. PTSD Checklist–Civilian Version (PCL-C) total score: green if ≤29, yellow to red from 30 to 85 (maximum possible score). 3. Pittsburgh Sleep Quality Index (Sleep) total score: green if ≤4, yellow to red from 5 to 24 (maximum possible score). 4. Neurological Examination: green if normal, red if abnormal (findings present on exam). 5. Sharpened Romberg Test (SRT): green if normal, red if abnormal (unable to perform to 30 seconds on any of 4 attempts). 6. Near Point of Convergence: green if ≤12.7 cm, red if >12.7 cm. 7. Brief Smell Identification Test (B-SIT): green if normal for age, red if abnormal for age. 8. Sustained Grip Strength: green if within 2SD of mean for age (70). 9. Heart Rate Variability (HRV): identified by subject matter expert as having abnormal HRV measures on 24-h Holter monitoring. 10. Electroencephalography (EEG): green if normal, red if abnormal. All abnormalities identified by clinical EEG testing in this population were generalized slowing. 11. Quantitative Electroencephalography (qEEG): green if normal, red if abnormal. 12. Eye Tracking: green if normal, yellow if abnormal performance on circular, horizontal ramp, or reading tasks 2 or 3 times over 3 testing intervals, red if abnormal 4 or more times over 3 testing intervals. 13. Vestibular: green if normal, yellow if identified by subject matter expert as having findings warranting clinical concern and further evaluation, red if identified by subject matter expert as having clinically abnormal vestibular testing. 14. Computed Tomography Angiography (CTA): cerebral blood flow and cerebral blood volume green if normal. Yellow if abnormal blood flow in 1 of 16 brain regions, red if abnormal in 2 regions (maximum observed). All abnormalities were were focal non-uniformities representing decreased arterial flow and volume. Regions were right and left frontal, parietal, temporal, occipital, basal ganglia, cerebellum, pons, and brain stem. 15. Overall MRI Impression: based on white matter lesion burden (clinical interpretation based on lesion count and size). Green if no lesions or lesions consistent with normal aging. Yellow if lesion burden greater than expected for age but unlikely to be seen at routine imaging. Orange if lesion burden greater than expected for age and likely to be seen at routine imaging. Red if severe/significant lesion burden. 16. Hippocampal and global atrophy: graded as normal (green), mild (yellow), moderate (orange), severe (red). 17. Cavum Septum, Size of Largest White Matter Hyperintensity, Encephalomalacia, Gliosis, Hypoxia/Ischemic Injury, Pineal Cyst, and Dilated Perivascular Spaces: graded as normal (green), tiny (0–3 mm) (yellow-green), small (4–6 mm) (yellow), medium (8–10 mm), large (>10 mm) (red). 18. Number of white matter hyperintensities. 19. Number of Regions with White Matter Hyperintensities: green if normal, red if abnormal in 7 of 19 regions (maximum observed). Regions were right and left frontal, parietal, temporal, occipital, cerebellum, corpus collosum genu, body, and splenium, midbrain, pons, and medulla. 20. Diffusion Tensor Imaging (DTI): green if normal, red if abnormal (fractional anisotropy and radial diffusivity >2 standard deviations outside the mean). 21. Empty Sella: green if normal, red if abnormal.

Figure 2 presents a participant-level distribution of the abnormalities found in this normal population. Generally, for the measures presented (selected to represent various functional domains), abnormalities were widely distributed across the population. A handful of participants were strikingly abnormal on many measures. Of interest, many domains expected to overlap did not. For example, there was no overlap between abnormal qEEG and clinical EEG interpretation. Similarly, abnormal eye tracking did not correlate with overall findings in the vestibular domain or with near point of convergence. Abnormal MRI did not appear to be associated with abnormal findings on other measures. Even participants with strikingly abnormal brain MRI had few or no clinical findings. When those with abnormal MRI, based on white matter lesion burden (50) or overall MRI impression, were compared to the rest of the group, they were not significantly more likely to express clinical abnormality (Table 10).

**Table 10:** Number and percent of participants with abnormalities by white matter lesion burden and overall MRI impression.

	**Total study** **group** **(*n* = 75)**	**White matter lesion burden**		**Overall MRI impression**	
		**Greater thanexpected for age(*n* = 22)**	**Consistent withnormal aging(*n* = 53)**	**“Clearlyabnormal”(*n* = 17)**	**“Clearlynormal”(*n* = 32)**
Neurobehavioral Symptom Inventory total score ≥11	3 (4%)	1 (5%)	2 (4%)	1 (6%)	2 (6%)
Pittsburgh Sleep Quality Index global score ≥5	21 (28%)	5 (23%)	16 (30%)	3 (18%)	9 (28%)
Obstructive sleep apnea risk	14 (19%)	4 (18%)	10 (19%)	3 (18%)	4 (13%)
Neurological examination	2 (3%)	1 (5%)	1 (2%)	1 (6%)	0 (0%)
Sharpened Romberg	16 (21%)	7 (32%)	9 (17%)	4 (24%)	6 (19%)
Near point of convergence >12.7 cm	37 (49%)	13 (59%)	24 (45%)	11 (65%)	15 (47%)
Brief smell identification test	2 (3%)	1 (5%)	1 (2%)	1 (6%)	1 (3%)
Grip strenth	21 (28%)	6 (27%)	15 (28%)	5 (29%)	7 (22%)
Heart rate variability	5 (7%)	1 (5%)	4 (8%)	1 (6%)	2 (6%)
EEG	6 (8%)	2 (9%)	4 (8%)	1 (6%)	4 (13%)
qEEG	18 (24%)	6 (27%)	12 (23%)	5 (29%)	6 (19%)
Eye tracking	19 (25%)	7 (32%)	12 (23%)	6 (35%)	7 (22%)
Vestibular function	16 (21%)	6 (27%)	10 (19%)	5 (29%)	3 (9%)
Cerebral blood flow	16 (21%)	4 (18%)	12 (23%)	3 (18%)	7 (22%)
Cerebral blood volume	17 (23%)	4 (18%)	13 (25%)	3 (18%)	8 (25%)
Hippocampal atrophy	8 (11%)	5 (23%)	3 (6%)	5 (29%)	2 (6%)
Global atrophy	2 (3%)	0 (0%)	2 (4%)	0 (0%)	0 (0%)
Cavum septum	33 (44%)	12 (55%)	21 (40%)	10 (59%)	12 (38%)
Encephalomalacia	2 (3%)	1 (5%)	1 (2%)	1 (6%)	0 (0%)
Gliosis	1 (1%)	0 (0%)	1 (2%)	0 (0%)	0 (0%)
Hypoxic/ischemic injury	1 (1%)	1 (5%)	0 (0%)	1 (6%)	0 (0%)
Pineal cyst	30 (40%)	11 (50%)	19 (36%)	10 (59%)	11 (34%)
Empty sella	12 (16%)	3 (14%)	9 (17%)	3 (18%)	4 (13%)
Dilated perivascular spaces	35 (47%)	12 (55%)	23 (43%)	10 (59%)	15 (47%)
Diffusion tensor imaging	2 (3%)	1 (5%)	1 (2%)	1 (6%)	1 (3%)

### Results of Subgroup Analyses

**Figure 3:**
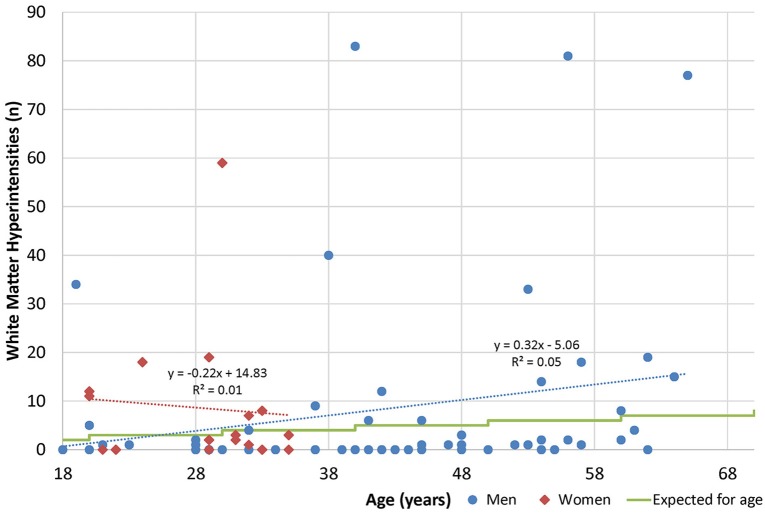
Age and white matter hyperintensities. Radiologists are commonly taught that one lesion per decade of life is considered normal (50).

By subgroup analysis, age and gender did have an effect over some measures (Table 11). For example, gender had an effect on standardized questionnaires at baseline (worse in men), but age did not. Men had better neurological function but worse sleep outcomes and quantitative neuroimaging, while older age was correlated with worse vestibular performance, sleep, and neuroimaging. Age and gender had less effect on changes over time, and age and gender were not associated with white matter hyperintensity burden (Figure 3).

**Table 11:** Age and gender subgroup analyses in selected domains.

**Domain**	**Gender effect at baseline**	**Age effect at baseline**	Gender effect over time^*^	Age effect over time^*^	**Significant change in univariate or post-*****hoc-tests***	
					**Gender subgroups**	**AGE subgroups**
Standardized questionnaires	Worse in males	No	No	No	No	Worsening in 36–45 subgroup
Neurological function	Better in males	Best in 46–55 age group, worst in 56–65 age subgroup	NPC^**^ only	NPC only	Improved NPC in males	Improved NPC in 36–45 subgroup
Vestibular/auditory (clinical interpretation)	Yes–direction of effects varies across outcomes	Generally worse in older age subgroups (46–55, 56–55)	Yes	Yes	Improvement in females	Changes (better and worse) most prevalent in older age subgroups
Visual systems (Eye tracker reading task)	No	Higher scores in 46–55 age subgroup	Minimal	Minimal	Some increases in males	Increases in 18–35 age subgroup
Autonomic function	Higher Holter measures in males in sleep Segment	Higher Holter measures in 56–65 subgroup (24 h, Awake)	24 h, Awake Segments	Yes, except Sleep Segment	Changes in males	Changes most prevalent in 56–65 age subgroup
Sleep	Worse in males	Worse in older age subgroups	No	No	No	No
Neuroimaging–qualitative	No	Worse in older age subgroups	No	No	No	No
Neuroimaging–quantitative	Worse in males	Worse in older age subgroups	No	Minimal	No	Some changes in Freesurfer most prevalent in 18–35 age subgroup

* *Overall difference between gender or age subgroups in changes over time*.

** *NPC–near point of convergence*.

### Safety

Because this was a non-interventional study, the definition of adverse events was limited to only those deemed to be related to study procedures (assessments). No participant experienced a serious adverse event during the study. Generally, the assessment battery was well-tolerated, including the 2.5-h-long MRI. Nearly half of adverse events were associated with the rigorous vestibular battery: 8 participants had nausea and/or vomiting, 3 reported dizziness, 2 had onset of headache, and 1 participant each experienced neck pain, fatigue, anxiety, and ear canal abrasion. Nine participants had skin irritation associated with Holter lead placement, and 5 experienced dizziness, vomiting, or hypotension in conjunction with the exercise segment of Holter monitoring. Three participants reported anxiety and 1 (age 29 years) reported vertigo with MRI. Three participants experienced a complication of IV placement for the CT scan (2 hematoma, 1 extravasation), and 1 developed a rash after contrast administration.

## Discussion

To our knowledge, this exploratory, observational study is the first to comprehensively evaluate normal, healthy volunteers across a variety of functional domains with a focus on measures of brain injury. Some measures used in this study, such as eye tracking, do not have sufficient published normative data available. Many measures used in this study have been tested in healthy populations (Table 12), but they have not necessarily been evaluated for stability over time, and very few have been correlated with measures in other functional domains. This study represents a unique effort to describe how a healthy population recruited from the community might perform on a wide variety of functional measures, and from that data, to better understand the “normal” brain. It also provides valuable information about changes over time in many of these measures.

**Table 12:** Sample of normal studies.

**Measure**	**Study**	**Summary**
Rivermead Post-Concussive Symptom Questionnaire	Thompson et al. (52)	RPQ administered to 46 healthy adults and 61 individuals with persistent post-concussion symptoms after mild-moderate TBI. Proposed cut-off scores: ≥16 for total score (97% sensitivity, 87% specificity).
	Iverson et al. (53)	Post-concussion symptom questionnaire similar to RPQ administered to 104 young, healthy individuals. At least 50% of participants reported mild headaches, fatigue, irritability, sad/down, nervous/tense, temper problems, poor concentration, memory problems, and poor sleep. At least 10% of participants reported moderate or severe fatigue irritability, temper problems, poor concentration, memory problems, and poor sleep.
Neurobehavioral Symptom Inventory	Belanger et al. (54)	Two hundred and fifteen active duty US military personnel completed NSI twice in 30 days. Depending on the endorsement level used, 2–15% met criteria for post-concussional disorder. Test-re-test reliability for total score was r = 0.78. An 8-point change in total score represented reliable change.
PTSD Checklist-Civilian version	Belanger et al. (54)	Two hundred and fifteen active duty US military personnel completed NSI twice in 30 days. Depending on the endorsement level used, 1–6% met criteria PTSD. Test-re-test reliability for total score was *r* = 0.70. A 7-point change in total score represented reliable change.
	Walker et al. (55)	One hundred and fifty two women with a history of trauma and 116 women with no history of maltreatment were interviewed and completed PCL. Optimal cut-off score was 30 (sensitivity 82%, specificity 76%).
Heart Rate Variability	Ewing et al. (56)	In 24-h ECG recordings collected from 67 healthy volunteers, significantly more HRV in younger than older participants
	Minassian et al. (57)	Of 2,430 active duty US military personal with 5-min HRV data, the 13% with PTSD had significantly lower HRV, even when adjusting for TBI history.
Pittsburgh Sleep Quality Index	Buysse et al. (31)	Fifty-two healthy and 116 sleep-disordered participants evaluated over 18 months. PSQI score >5 (poor sleep) yielded diagnostic sensitivity of 90% and specificity of 87%.
	Mollayeva et al. (58)	Meta-analysis of 22 studies that administered PSQI to non-clinical participants. Mean global PSQI for non-clinical participants ranged from 2.7 to 6.7.
Electroencephalography	Gschwandtner et al. (59)	Pathological slowing in 4/35 healthy controls (11%), compared to 22/72 patients (31%).
	Sachdev et al. (60)	In 33 controls (age >50), 4 (13%) had slow waves, 3 (9%) atypical sharp waves, 4 (14%) paroxysmal activity, 2 (6%) focal activity, and 1 (3%) generalized slowing.
Sharpened Romberg Test	Lee et al. (28)	In 53 non-diving volunteers and 48 divers, 95% were able to achieve the Sharpened Romberg for 60 s by the fourth attempt. However, attempts that lasted <5 s were considered false starts and not counted.
Near Point of Convergence	Abraham et al. (61)	In 100 young adults (ages 19–35), the 95% confidence interval for objective near point of convergence was 7.75–9 cm.
	Scheiman et al. (62)	In 175 optometry students (ages 22–37), mean near point of convergence was 2–2.5 cm, depending on the method tested, with an upper limit of 7–11 cm.
Six Minute Walk Test	Casanova et al. (63)	In 444 healthy adults from 7 countries, men walked a mean 30 m greater than women. Mean ± 1SD distances were: 40–49 years of age, 611± 85 m; 50–59 years of age, 588 ± 91 m, 60–69 years of age, 559 ± 80 m; 70–80 years of age, 514 ± 71 m.
Hand Grip Strength	Massy-Westropp et al. (64)	In 1,314 men and 1,315 women, mean grip strength declined in the 50+ years age groups. Mean ± 1SD grip strength for younger men was approximately 47 ± 10 kg and 28 ± 6 kg for younger women.
Ocular Torsion	Lee et al. (65)	In 100 opthalmologically normal participants, the angle of ocular torsion was 6.11 ± 3.21° in the right eye, 6.67 ± 3.18° in the left, and the mean was 6.39 ± 3.20°. Age and sex were not significantly associated with ocular torsion.
Dynamic Visual Acuity	Honaker et al. (66)	In 89 healthy adults age 20–79 years, gaze stabilization and dynamic visual acuity singificantly decreased with age, and perception time increased with age.
Computerized Dynamic Posturography	Hageman et al. (67)	In 24 healthy adults (20–35 years and 60–75 years), older adults had larger sway on all the six test conditions, along with longer movement times, longer path lengths, and shorter functional reach distance. There were no differences in performance between men and women.
Rotational Chair Testing	Akin et al. (68)	In 24 healthy young adults, subjective visual vertical test was <2 degrees for static and on-axis rotation but shifted up to 11 degrees during unilateral centrifugation.
Neuroimaging	McGuire et al. (69)	In 82 doctorate-degree controls, a high resolution 3T MRI revealed 3.3 ± 4.5 (mean ± 1SD) white matter hyperintensities, with a mean volume of 0.04 ± 0.07 cm^3^.
	De Perri et al. (70)	Fifty-three healthy controls (18–63 years old) underwent 1.5T and 3T MRI. The median individual white matter hyperintensity volume was 68.5 mm^3^ (range 0–752.6) on 1.5T and 374.4 (range 0–2,460) on 3T.
	Riedy et al. (50)	In 42 controls without TBI undergoing 3T MRI, 38% had cavum septum, 57% had dilated perivascular spaces, and 38% had white matter hyperintensities. These rates were not significantly different from the incidence in 834 TBI participants. However, only 1 of 42 controls had more than 1 lesion per decade of life, compared to 22% of TBI participants
	Neema et al. (71)	Twenty-two healthy controls (30–53 years old) underwent 1.5 and 3 T MRI. Particiapnts had a mean 5.5 ± 9.1 discrete hyperintense foci (range 0–33) by 1.5 T and 10.7 ± 14.4 (range 0–47) by 3 T.
	Pu et al. (72)	In 100 young, healthy adults (ages 19–39 years old) undergoing 1.9 T MR imaging, 23 had pineal cyst (>2 mm), and 13 had small cystic changes (<2 mm).
	Sun et al. (73)	In 112 healthy volunteers (mean age 25), 25% had pineal cyst or cystic changes.
	Hopkins et al. (51)	243 healthy volunteers (16–65 years old) with 1.5 T MRI. Only 2.5% of participants ≤55 years old had any white matter hyperintensities, compared to nearly 25% of those >55 years old. No participant in their 40's had white matter hyperintensities.

Contrary to what one might expect, we found abnormalities dispersed across the study population (Figure 2). The number of abnormalities may be a function of the large number of tests that these participants underwent, in that had they undertaken fewer tests, there would likely be fewer findings. This suggests that some number of healthy individuals may be expected to have abnormal performance on any given measure at any time.

Direct comparisons of results between other “normal” studies is challenging. Often, normative values are collected for the purpose of comparison to patients with a specific disease or condition, and “healthy” or “normal” are defined as the lack of that disease or condition. Because these participants were intended to be compared to military personnel with mild TBI, we focused on screening out brain injury and conditions that might manifest similar to mild TBI. Other studies that have collected normative data may have enrolled participants who differ in significant ways from participants in this study. In addition, differences in equipment, personnel, and administration and scoring methodology can confound attempts to directly compare normative values from one study to another.

A handful of participants in this study appeared to have clusters of abnormalities and may have underlying brain dysfunction, possibly due to prior brain trauma, though we did not establish a threshold for determining what might represent brain injury beyond screening for TBI and other brain injuries using validated instruments. The study had strict enrollment criteria, and any history of brain insult was an absolute exclusion for participation. Participants underwent multiple layers of screening before they were assessed, yet, based on outcome data, it appears that a few participants with possible brain injury joined the study. These individuals may truly have no history of brain injury, they may have had no recollection of prior brain injury, or they may have been disingenuous during screening procedures in order to be compensated for participation. Nevertheless, these participants likely comprise a small minority of the study group and do not account for the abnormalities that are distributed across many study participants.

It is possible that our enrollment criteria were insufficiently strict to exclude all individuals with brain dysfunction. In designing this study, we considered requiring all participants to have a normal screening brain MRI. However, we felt this requirement would select “supernormal” individuals that would not represent a true normal population. Had we required a normal screening MRI, nearly 60% of our study group would have been excluded. However, this requirement would not have necessarily reduced the frequency of abnormalities in other domains (Table 10).

When comparing our enrollment criteria to studies recruiting normal volunteers, particularly for brain imaging, our criteria were more stringent. For example, one component of the Human Connectome project recruiting healthy volunteers allows individuals with up to 2 lifetime mild TBIs or a history of substance abuse (without severe symptoms) to participate (74). Another component of this project (NCT02193425) recruiting healthy volunteers allows head trauma with loss of consciousness up to 30 min, and volunteers with positive urine drug screens are invited to return for scanning after a few days. Whether these methods can more reliably enroll individuals with “normal” neuroimaging is unknown.

Despite the number of individual abnormalities discovered, this study's participants, as a group, differentiated from the group of individuals with mild TBI who underwent the same evaluations. For example, abnormal facial sensation, tandem gait, tremor, and Sharpened Romberg were more common in the mild TBI group, as were generalized and localized slowing on EEG. Similarly, group mean data for HRV parameters (16), sleep measurements (75), and eye tracking measures (76) were significantly different between the two groups.

The number and degree of abnormalities noted on neuroimaging in this study was unexpected. In our study, participants were scanned on a 32-coil 3.0 Tesla MRI with 1 mm sections, and this high resolution may have allowed more neuroimaging abnormalities to be identified. White matter hyperintensities are a non-specific finding associated with trauma (77), carbon monoxide poisoning (78), hypoxia (79), microvascular disease [as in diabetes mellitus (80)], illicit drug use (81), and the aura form of migraine (82). This study excluded all these populations based on participant self-report, and laboratory testing was negative for diabetes mellitus and illicit drug use. Yet, our results (25 of 65 participants (38%) ≤55 years old with at least 2 white matter hyperintensities) stand in contrast to other work reporting the prevalence of white matter hyperintensities in healthy individuals as 5.3%, with increased numbers of lesions in those age ≥55 years old (51), though this prior study was performed on a 1.5 Tesla scanner.

Untreated hypertension may be associated with white matter hyperintensities in the elderly (83). Four participants in the older age group were receiving medical therapy for hypertension, and 3 had more lesions than expected for age. The highest blood pressure reading recorded during this study (152/87 mmHg) occurred in a 62-year-old man with no white matter hyperintentisities, and because this measurement did not follow current best practice guidelines, its clinical significance is unclear. Obstructive sleep apnea may increase the risk for white matter changes independent of its contribution to hypertension (84). Our study participants were recruited from a locale 6,000 feet (1,840 m) above sea level, and increased altitude is associated with sleep disordered breathing in healthy adults and worsened sleep apnea in patients (85). We did not perform nighttime oximetry or polysomnography to screen for or diagnose sleep apnea, but 14 participants had high or intermediate risk for obstructive sleep apnea by STOP-Bang; however, this measure was not associated with MRI findings.

In addition to assessing the prevalence of abnormalities in healthy volunteers, another purpose of this study was to measure changes over time in a population that should be relatively stable. The standardized questionnaires administered in this study exhibited strong temporal reliability, as did the visual systems assessments (dynamic visual acuity by ETDRS chart, retinal fundoscopy, and eye tracking, except the reading task), and neuroimaging. In contrast, most vestibular, auditory, autonomic, and neurological function measures (near point of convergence and Sharpened Romberg test) were more variable over time.

The primary limitation to this study is the relatively small sample size, particularly given the large number of outcome measures and the interest in age/gender subgroup analysis. The study's sample size was determined according to estimates provided in the literature on detecting signal on quantitative neuroimaging measures; however, the assessment battery also included over 100 other outcomes across multiple domains. While this study enrolled more participants than many studies of normal volunteers (Table 12), the complexity and number of measurements would likely require a much larger sample size to estimate the true rate of abnormalities or to detect differences among subgroups in adults without brain injury across this substantial number of outcomes. However, a larger sample size was limited by available personnel and equipment resources, the geographic recruitment pool, and budgetary constraints.

The high rate of abnormalities observed on some measures may suggest the prevalence of these findings in the general population could be higher than anticipated, or it may suggest that our specific population had underlying brain dysfunction, which would limit the degree in which our results generalize to a truly healthy population. Regardless, a much larger study would be needed to define the base rate of abnormalities in the general population. In addition, fewer women were enrolled so that we could better match the brain-injured military population enrolled in the companion interventional studies for persistent post-concussive symptoms, and therefore information about older women is lacking.

Whether our normative data extrapolates to any other normal population is unknown. In our study, the mean age was 39, other normative populations may not be age matched or education matched. It is possible that some of the questionnaires could be influenced by age and education but we are underpowered to address those specific subgroups.

Additional study limitations include recruiting participants from a single metropolitan area. While the single assessment site brings standardization in equipment and methodology, there may be features of the study population that are not generalizeable. The significant time commitment required from participants and the level of compensation may have biased both recruitment and study results. The study was conducted at increased altitude and in a state where recreational marijuana use is legal, and nearly 10% of potential participants were excluded based on marijuana or illicit drug use, which may have influenced the composition of the study population. However, no participants in the analysis population had positive drug screens during study participation. An additional limitation is the omission of formal neuropsychological testing, which was not done because our anticipated enrollment into this exploratory study was relatively small, and norms for these tests are well-established from larger studies. In retrospect, an assessment of neuropsychological performance would have provided a more complete clinical picture of this study population.

For clinicians caring for individuals with brain injury, we recommend being circumspect about the results of this study compared to the results of other studies of healthy volunteers. While this study incorporated a prospective design and comprehensive, multi-domain assessments and represents a unique, concerted effort to establish normal brain function, its results are at odds with much of the other literature. Whether these results extrapolate to other populations is truly unknown, but we believe that rejecting abnormalities discovered in patients with a clinical history of brain injury as normal variants is not justified by this study's results.

This study was designed to recruit participants with no history of brain injury, and the results of this paper may be most valuable as a comparator to TBI studies (16, 75, 76) than for use as broadly generalizeable population norms. Ultimately, our results demonstrate that defining a “normal” population is challenging. Nevertheless, when paired with results in individuals with mild TBI undergoing the same tests, using the same equipment, personnel, and facilities, these studies provide important information about the differentiation between normal, healthy individuals and those with persistent post-concussive symptoms following mild TBI.

## Ethics Statement

This study was conducted in accordance with the International Conference on Harmonization guidelines for Good Clinical Practice and the Declaration of Helsinki. In the conduct of research where humans are the subjects, the investigator(s) adhered to the policies regarding the protection of human subjects as prescribed by Code of Federal Regulations (CFR) Title 45, Volume 1, Part 46; Title 32, Chapter 1, Part 219; and Title 21, Chapter 1, Part 50 (Protection of Human Subjects). The NORMAL study was approved by the United States Army Medical Research and Materiel Command Institutional Review Board; written informed consent was obtained for all participants prior to administering study assessments. The views, opinions and/or findings contained in this report are those of the author(s) and should not be construed as an official Department of the Army position, policy or decision unless so designated by other documentation.

## Author Contributions

All the authors vouch for the accuracy and completeness of the data and data analyses and for the fidelity of the trial to the protocol. SW and AL performed the data analysis. LW and KD prepared the first draft of the manuscript. LW, SW, AL, SC, KD, RP, CW, WO, JP, JW, AM, and SM participated in the writing of the manuscript and approved the draft that was submitted for publication. The results were reviewed by the Sponsor.

### Conflict of Interest Statement

All authors received salary support from the study Sponsor (United States Army Medical Research and Materiel Command) through their employers, except RP, who was active duty for the duration of this study.
